# The Micronesia Challenge: Assessing the Relative Contribution of Stressors on Coral Reefs to Facilitate Science-to-Management Feedback

**DOI:** 10.1371/journal.pone.0130823

**Published:** 2015-06-18

**Authors:** Peter Houk, Rodney Camacho, Steven Johnson, Matthew McLean, Selino Maxin, Jorg Anson, Eugene Joseph, Osamu Nedlic, Marston Luckymis, Katrina Adams, Don Hess, Emma Kabua, Anthony Yalon, Eva Buthung, Curtis Graham, Trina Leberer, Brett Taylor, Robert van Woesik

**Affiliations:** 1 University of Guam Marine Laboratory, UOG Station, Mangilao, Guam; 2 Commonwealth of the Northern Mariana Islands Bureau of Environmental and Coastal Quality, Saipan, Marianas Protectorate; 3 Conservation Society of Pohnpei, Kolonia, Pohnpei State, Federated States of Micronesia; 4 Kosrae Conservation and Safety Organization, Lelu, Kosrae State, Federated States of Micronesia; 5 Kosrae Village Resort, Malem, Kosrae State, Federated States of Micronesia; 6 College of the Marshall Islands, Majuro, Republic of the Marshall Islands; 7 Marshall Islands Marine Resources Authority, Majuro, Republic of the Marshall Islands; 8 Yap Community Action Program, Colonia, Yap State, Federated States of Micronesia; 9 Chuuk Marine Resources Department, Weno, Chuuk State, Federated States of Micronesia; 10 The Nature Conservancy Micronesia Program, Guam Field Office, Hagatna, Guam; 11 James Cook University, Townsville, Australia; 12 Department of Biological Sciences, Florida Institute of Technology, Melbourne, Florida, United States of America; University of California Santa Cruz, UNITED STATES

## Abstract

Fishing and pollution are chronic stressors that can prolong recovery of coral reefs and contribute to ecosystem decline. While this premise is generally accepted, management interventions are complicated because the contributions from individual stressors are difficult to distinguish. The present study examined the extent to which fishing pressure and pollution predicted progress towards the Micronesia Challenge, an international conservation strategy initiated by the political leaders of 6 nations to conserve at least 30% of marine resources by 2020. The analyses were rooted in a defined measure of coral-reef-ecosystem condition, comprised of biological metrics that described functional processes on coral reefs. We report that only 42% of the major reef habitats exceeded the ecosystem-condition threshold established by the Micronesia Challenge. Fishing pressure acting alone on outer reefs, or in combination with pollution in some lagoons, best predicted both the decline and variance in ecosystem condition. High variances among ecosystem-condition scores reflected the large gaps between the best and worst reefs, and suggested that the current scores were unlikely to remain stable through time because of low redundancy. Accounting for the presence of marine protected area (MPA) networks in statistical models did little to improve the models’ predictive capabilities, suggesting limited efficacy of MPAs when grouped together across the region. Yet, localized benefits of MPAs existed and are expected to increase over time. Sensitivity analyses suggested that (i) grazing by large herbivores, (ii) high functional diversity of herbivores, and (iii) high predator biomass were most sensitive to fishing pressure, and were required for high ecosystem-condition scores. Linking comprehensive fisheries management policies with these sensitive metrics, and targeting the management of pollution, will strengthen the Micronesia Challenge and preserve ecosystem services that coral reefs provide to societies in the face of climate change.

## Introduction

Micronesia is comprised of a suite of tropical island nations that together amount to more than 3,000,000 km^2^ of the north Pacific Ocean, with over 6,000 km^2^ of coral reefs [1]. This region is home to island societies that have coexisted with marine resources for generations under traditional-tenure systems [2,3]. Yet growing influences of human-population expansion and cash-based economies have begun to erode the traditional forms of sustainable reef management. These changes have increased pressure upon marine resources [4,5]. In addition, recent climate-change-related increases in sea-surface temperatures, ocean acidification, and extreme weather patterns, which are largely attributable to carbon dioxide emissions from developed nations, have begun to shorten the timeframe between disturbances on reefs [6–8]. Some examples of influential disturbances include: (i) increased frequencies of high-temperature anomalies that lead to coral bleaching [9] (ii) high-intensity storms that increase watershed-pollution discharge into adjacent marine environments [10,11], and (iii) the combined impacts of watershed runoff, shifting winds and upwelling in the tropical Pacific Ocean that are associated with population outbreaks of the coral predator *Acanthaster planci* [12–15]. The increased frequencies of acute disturbances, and their interaction with chronic stressors from local-pollution sources, have already changed the species composition on many reefs [16–18].

Although small-island nations have little control over greenhouse gas emissions from developed nations, they can however manage their local resources to enhance the ecosystem services that the reefs provide [19,20]. In this spirit, the political leaders of five nations in Micronesia initiated a friendly challenge across jurisdictions in 2006 to ‘effectively conserve’ at least 30% of their marine resources and 20% of terrestrial resources by 2020 [21]. The political leaders of Micronesia realized that their island societies depend on sustainable fishery yields and other ecosystem services that are provided by healthy reefs. The initiation of the ‘Micronesian Challenge’ was novel because it originated with a strong political will to preserve the local environment at a regional scale. Such an initiative is often problematic across political boundaries because science-to-management frameworks often become decoupled at large spatial scales. The implementation of the Micronesia Challenge empowered scientists and managers to provide the necessary information on reef condition and current threats in order to develop an optimal conservation strategy. Yet, the scientific basis for disentangling localized stressors is still emerging.

Although it is well established that coral reefs are comprised of diverse species networks across multiple trophic guilds, it is important to assess whether managing for diversity is essential, and to what extent diversity can help maintain ecosystem services through disturbance cycles. Since the challenging hypothesis generated by Robert May purporting no reason for diversity to generate ecosystem stability [22], ecologists have come to appreciate that diversity can be linked with ecosystem stability (i.e., rate of return to a desirable, functional state following a perturbation), but only within certain contexts. First, species interactions should be comprised of many weak links, representing satellite species with high ecological redundancy, and of few strong links, representing species that often act as ecosystem engineers (i.e., species interaction strengths are coupled with species abundance patterns) [23,24]. Second, standing-stock estimates of biomass within food webs should be disproportionally retained within slow energy-transfer channels compared with fast energy-transfer counterparts, as the latter rapidly cycle to fuel the sparse but strong trophic linkages [25,26]. Third, the distribution of species interactions should exist as repeated food-web motifs that serve to maximize ecosystem productivity, while maintaining stable population dynamics among functional trophic guilds [27,28]. It follows that diversity within trophic guilds, such as herbivorous fishes, may provide a high functional redundancy, while dampening the oscillations of individual species population cycles [29]. By contrast, the loss of diversity, reduced abundances of ecosystem engineers, or loss of keystone predators may result in altered trophic relationships that underpin the patterns of reef decline observed along gradients of human influence [30].

To examine the effects of human populations on the diversity, function, and status of coral-reef ecosystems across Micronesia, this study: (i) assessed ecosystem condition across six jurisdictions to evaluate the conservation goals of the Micronesia Challenge; (ii) examined the distribution and variance of ecosystem condition within major reef habitats as indicators of ecological stability, and (iii) considered the role of two putative stressors in driving ecosystem condition—fishing and pollution. We defined ecosystem condition using a series of biological metrics that were related to reef states, reef processes, and several ecological principles (e.g., density and size of herbivores, herbivory, and allometric-scaling laws that define grazing rates, S1 Table). By contrast, fishing pressure and pollution were derived from abiotic metrics of wave exposure, land-use, and distances to fishing access and pollution discharge. Coupling biological metrics with their associated environmental regimes improved our ability to assess the individual contributions of localized stressors to ecosystem dynamics, with outcomes that were relevant for optimizing both local and regional management efforts.

## Methods

### Ethics Statement

Data were collected by all authors in collaborative partnerships. Cumulatively, the organizations involved in data collection were the Marshall Islands Marine Resources Authority, Marshall Islands Conservation Society, College of the Marshall Islands, Kosrae Conservation and Safety Organization, Kosrae Island Resource Management Authority, Conservation Society of Pohnpei, Pohnpei State Fisheries, Chuuk Conservation Society, Chuuk Marine Resources, CNMI Bureau of Environmental and Coastal Quality, Yap Community Action Program, and Yap State Fisheries. These organizations are responsible for coral-reef monitoring activities and have the authority to conduct research. Further, non-invasive research was conducted which included photographs and visual estimates described in the methods. Given the non-invasive nature of research, no permits were required. Supporting information contains the GPS coordinates for all research sites.

**Fig 1:**
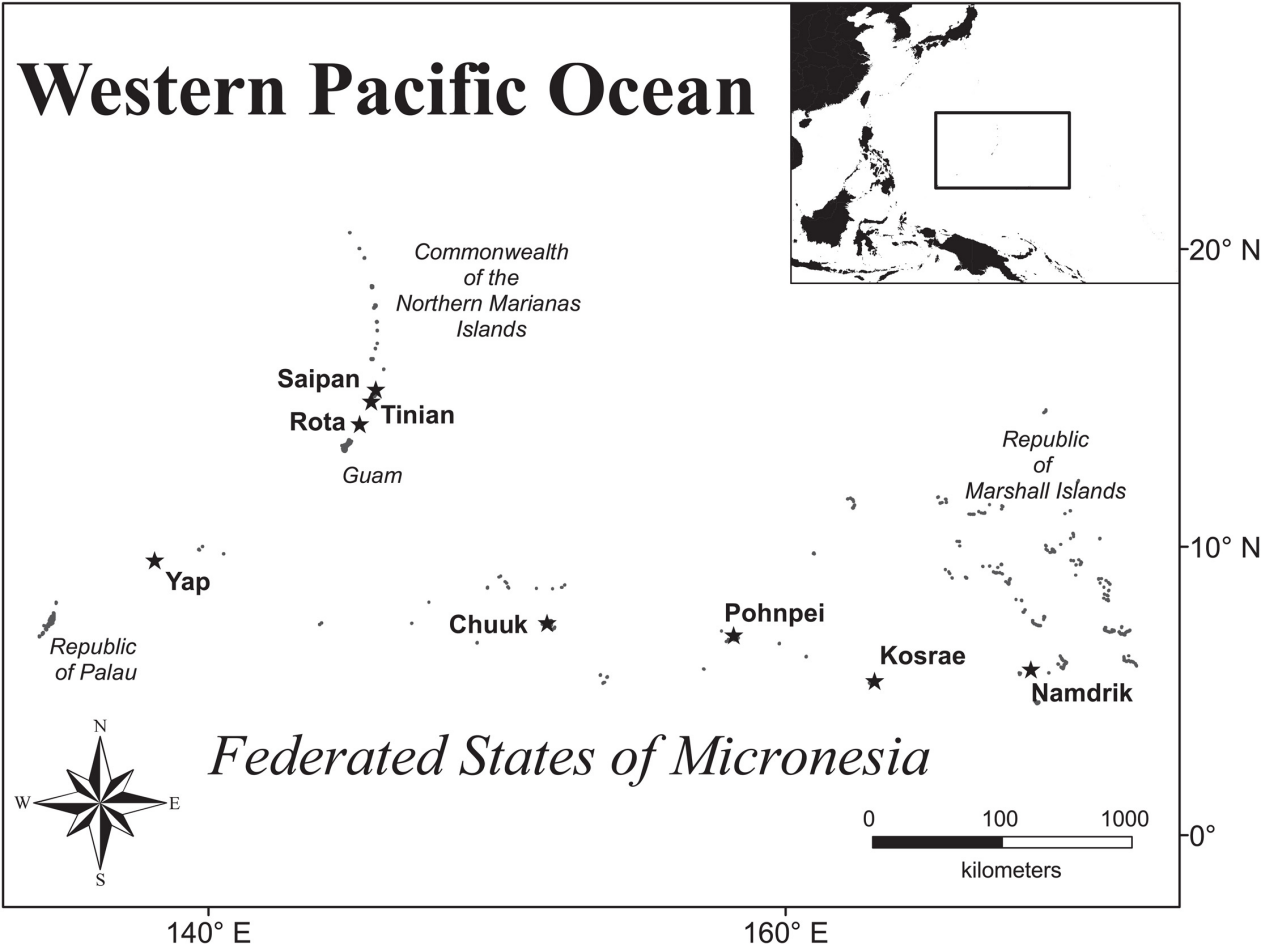
A map of the tropical Western Pacific Ocean and Micronesia with study islands indicated by stars.

The present study examined the coral reefs in six jurisdictions across Micronesia: (i) the Marshall Islands, the states of (ii) Kosrae, (iii) Pohnpei, (iv) Chuuk, and (v) Yap, which comprise the Federated States of Micronesia, and (vi) Commonwealth of the Northern Mariana Islands (Fig 1). Each jurisdiction was a signatory on the 2006 declaration establishing the Micronesia Challenge (MC) [21]. Given that the intent of the MC incorporates social well-being, the first assumption made by the regional MC scientific-measures team was that ‘effective conservation’ should be evaluated on the main islands of each jurisdiction, centered near human populations. The present study also took advantage of a recent coral-reef assessment conducted for Namdrik Atoll, Marshall Islands, which was used as a reference site for the present study because the atoll has a relatively low human population and has developed an award-winning, sustainable management program for its marine resources [31].

The reef-assessment protocol was designed to address the highest priority management questions at both local and regional scales (S1 Fig). Accordingly, sites were stratified across (i) management regimes, (ii) wave exposure, (iii) islands, and (iv) major reef habitats, to be representative of each island (S1 Table). Cumulatively, the present study included data from 78 sites across Micronesia, which were examined between 2012 and 2015 (S2 Table).

Field protocols were designed with high-statistical power (80%) to detect a relative change of 25% for all the benthos that supported absolute abundances of 20% or greater at the site level [32]. At each site, five 50-m transects were used to measure fishes, corals, and other benthic assemblages between 8–10 m on outer reefs, and at 3–5 m for inner lagoon reefs. A single depth was selected to maximize the number of monitoring sites across the region, while keeping within logistical constraints. Depths were selected to match zones of optimal coral growth.

Benthic substrates were evaluated using a photo-quadrat technique. Fifty photos were taken at 1-m intervals along each 50-m transect line. Within each photo the benthic substrates were evaluated under five randomly allocated crosses. Benthic-assemblage metrics were derived from data aggregated across the 50 photographs, to the transect level (n = 5). Metrics included coral and macroalgal cover, coral-genus richness, and a benthic substrate ratio defined by the percentage cover of heavily calcifying (corals and CCA) versus non-or-low calcifying (turf, encrusting, and macroalgae) substrates.

Coral-assemblage data were collected by a single observer (PH) in all of the jurisdictions except CNMI, where a calibrated observer (SJ) also collected data. During each survey, 10 replicate 1 m^2^ quadrats were haphazardly tossed at equal intervals along the transect lines. On the reefs of CNMI, 16 tosses of a 0.5 m^2^ were alternatively used to account for inherent difference in coral assemblages. Sampling intensity was selected in accordance with multivariate data saturation points, whereby individual quadrats were sequentially incorporated into principle component ordinations until a saturation point was exceeded [32,33]. For each quadrat, all coral colonies with their center points within the quadrat boundary were measured for maximum diameter (*x*), and for the diameter perpendicular to the maximum (*y*). Surface area was calculated assuming colonies were elliptical. Coral taxonomy followed [34].

The size and abundance of fishes, which are generally consumed by people (hereinafter food-fish), were collected by four calibrated observers, with individual observers being consistent across jurisdictions. Fish assemblages were estimated from 12 stationary-point counts (SPCs) conducted at equal intervals along the transect lines. At each SPC, the observer recorded the species name and the size of all food-fish within a 5 m circular radius for a period of 3 minutes. Food-fish were defined as acanthurids, scarids, serranids, carangids, labrids, lethrinids, lutjanids, balistids, kyphosids, mullids, holocentrids, and sharks. The sizes of fishes were binned into 5 cm categories, and converted to biomass using coefficients from regional fishery-dependent data when available, or from FishBase (www.fishbase.org).

### Classification of reef habitats

The major reef habitats were distinguished using principle component analyses, ordination plots, and multivariate tests of comparison using the benthic-substrate data [35]. Subsequent analyses of ‘ecosystem condition’ were nested within both jurisdictions and major-reef habitats. The reef habitats differed in accordance with island geology, however some habitats were common throughout the region, including outer reefs, inner reefs, patch reefs, and channel reefs. In the case of CNMI, previous work had already identified the main reef types, including spur-and-groove reefs, interstitial-framework reefs, and Rota-island reefs with limited Holocene framework [36]. Notably, these reef types had a non-uniform distribution across CNMI. Only outer reefs were examined on Namdrik and Kosrae.

### ‘Effective management’ and ‘ecological condition’

**Fig 2:**
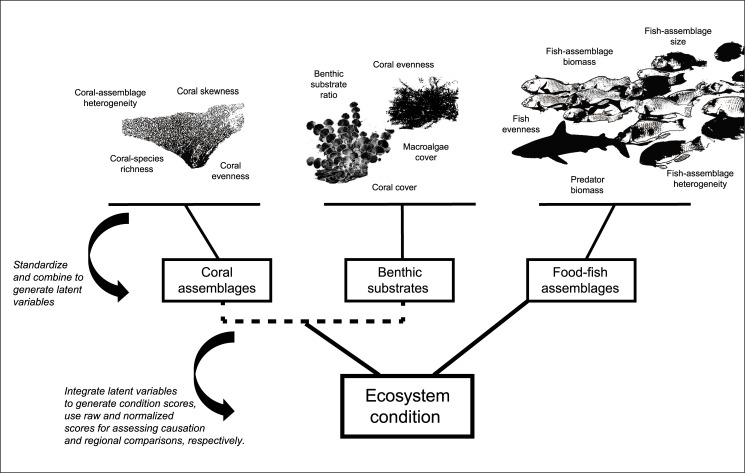
Ecosystem condition evaluation process depicting the contribution of individual biological metrics on their respective latent variables, and the weighting of latent variables on overall condition scores. Latent variables were generated from independent datasets. Biological metrics were established by a working group of regional scientists, and linked with key processes required for ecosystem function and maintenance (S1 Table).

In order to assess ‘effective management’ and ‘ecological condition’, latent variables were generated from each of the biological data outlined above (Fig 2). Latent variables were comprised of several biological metrics that originated from working groups across the jurisdictions. Metrics were associated with ecological processes and principles central to ecosystem function and maintenance (S1 Table). While attention was given to omit highly correlated metrics, sensitivity analyses were performed to assess their relative contributions. Both the diversity of latent variables and stratification by major habitats within each jurisdiction were used to reduce anomalous influences of past disturbances on estimates of ecological condition. Major disturbances have not been observed on the study islands since 2009 with the exception of Kosrae, where a moderate bleaching event occurred in 2013 (personal observation, PH), two years prior to data collection. Elsewhere, data were collected before recent thermal-stress anomalies in CNMI and Marshall Islands.

Latent variables associated with coral assemblages were derived from coral-colony surveys: (i) assemblage heterogeneity, (ii) size-frequency skewness, (iii) species richness, and (iv) Shannon-Weaver evenness. Heterogeneity was measured by multivariate Bray-Curtis dissimilarities, which were highest with species composition differences among replicate coral quadrats [37]. The skewness of size-frequency distributions was an approximate measure of the dominance of specific coral-colony sizes [38]. Latent variables associated with benthic substrates were derived from photo-quadrat surveys: (v) coral cover, (vi) benthic-substrate ratio, (vii) coral evenness, and (viii) macroalgal cover. Latent variables associated with the food-fish assemblages were: (ix) assemblage heterogeneity, (x) fish-assemblage size, (xi) fish-assemblage biomass, and (xii) apex-predator biomass.

All metrics were sorted from low-to-high and standardized by reef type within each jurisdiction, with reef types within islands herein referred to as strata. Ecosystem-condition scores were calculated using the following equation:

$$\frac{(\frac{c_{i}+b_{i}}{2})+f_{i}}{2}$$ (1)

where c_i_, b_i_, and f_i_, represent latent-variable scores for corals, the benthic substrate, and food-fish, respectively, for each site (*i*). These calculations provided equal weighting to the mobile and sessile components of the ecosystem. Ecosystem-condition scores were normalized between 0 and 100%, and ‘effective conservation’ was attributed to all sites that were within one standard deviation (mean SD = 30%) of the maximum score. Effective conservation for the Micronesia Challenge was then calculated by taking the number of sites that exceeded the 70% threshold. The logic behind establishing a cutoff threshold was to facilitate a regional comparison that highlighted the relative differences among jurisdictions and habitats. Yet, complementary investigations between ecosystem-condition scores and localized stressors were conducted and should be considered alongside condition scores.

Sensitivities of individual metrics were evaluated by examining the range of Pearson’s moment correlations with corresponding latent variables. Correlation matrices were also created from the constituents of all latent variables to understand their relationships. Ecosystem stability, or the stability of current ecosystem-condition scores, was estimated by the variance associated with the top four ecosystem condition scores within each stratum. High variances suggested low stability, because if any particular reef that is contributing towards effective-conservation thresholds declines in the future, then the probability is low that a different reef will improve and replace the depauperate reef.

### Predictors of ecosystem condition

Proxies of fishing pressure and land-based pollution were evaluated for their ability to predict ecosystem-condition scores. Both variables represent putative, localized stressors across Micronesia reef ecosystems. These proxies were derived from wave-energy statistics, land-use data, and distances to both main fishing ports and watershed discharge. Site-based wave energies were calculated based upon 10-year wind-speed records, fetch distances to the nearest reef or land feature, and angles of exposure (Quikscat wind datasets from 1999 to 2009; https://winds.jpl.nasa.gov/, wave energy in J/m^3^, full description found in [20, 39–40]. The proxy for fishing pressure was calculated by multiplying standardized wave energies and distances to the nearest points of fishing access (i.e., wind and distance interactions, S2 Table). A proxy for land-based pollution was developed from United States Geological Survey topographic maps and United States Forest Service land-use data (United States Forest Service, http://www.fs.usda.gov/r5). The proxy for pollution was calculated by summing the coverage of barren land, urbanized vegetation, and developed infrastructure within each watershed, and multiplying that sum by the distance to the primary-discharge point (i.e., altered land and distance interactions). For the pollution proxy, distances were inversely scaled to match intuitive low-to-high influences. For the fishing-pressure proxy, both wave exposure and distance to fishing ports were both inversely scaled. Proxies were standardized within each stratum to match the biological data.

Generalized linear mixed-effect modeling was performed, using a nested design (analysis of covariance), to examine the extent to which abiotic proxies predicted ecosystem-condition scores. Although localized stressors were standardized, we were careful not to assume that localized-stressor gradients were consistent across islands and across reef types (i.e., distances between y-intercepts or slopes). We compared mixed-effect models that included random error terms to account for potential differences across the region. Statistical modeling was performed using ‘lme4’ package in R [41]. Resultant models were examined for residual normality, using Shapiro-Wilk tests, and evaluated by their explanatory power, confidence intervals, and p-values.

Prior to statistical modeling, the experimental strata were grouped into two main subsets of reef habitats based upon their physical setting: 1) outer, patch, and channel reefs that were situated > 5 km from major land-based discharge, with reef structures or lagoon islands blocking the land-based discharge from the reefs, and 2) inner and channel reefs that were within 5 km of major watersheds. Because the magnitude of localized stressors differed across strata, we also examined whether the slopes (*β*) of independent relationships could predict ecosystem stability, or variance in ecosystem-condition scores, using least-squares regression modeling in R. The presence of outliers in the analyses were either removed or conditionally examined when components of fishing pressure and pollution proxies, such as wave exposure and disturbed land, had values greater than 2 standard deviations above the mean (e.g., Site 7, CNMI). Several no-take marine protected areas existed across Micronesia (S2 Table); while we recognized their potential influence, we did not assume they supported comparatively higher ecological metrics than areas outside of protected areas. Instead, we examined models with and without marine protected areas for best-fit. To further understand the hypothesized relationships between ecosystem condition and localized stressors we examined correlations between the individual constituents of the abiotic fishing pressure proxy, wave energy and distance to boat access, and the biotic components of ecosystem condition, benthic, coral, and fish latent variables, for any improved association over integrated proxies or latent variables.

## Results

Inherent differences in coral and benthic-substrate abundances existed across major reef habitats in Micronesia primarily because of wave exposure and proximity to watersheds (S2 Table and S2 Fig). In Yap, benthic assemblages were most distinctive across inner, channel, and outer reefs (PERMANOVA pairwise t-statistics > 3.0 for all, P<0.001). Similarly, channel, patch, and inner reefs were distinctive in Chuuk (t-statistics > 1.62 for all, P<0.01). In Pohnpei, inner and outer reefs were distinctive (t-statistic = 2.95, P<0.001), yet within the inner lagoon, patch, and fringing reefs were similar, which was likely a characteristic of island geology and deep lagoon waters yielding similar wave exposure (S2 Table). Combined with previous studies in CNMI, which also identified distinctive reef habitats, this study utilized 12 strata to examine ecosystem condition and stability, and their association with potential stressors (S2 Table).

**Fig 3:**
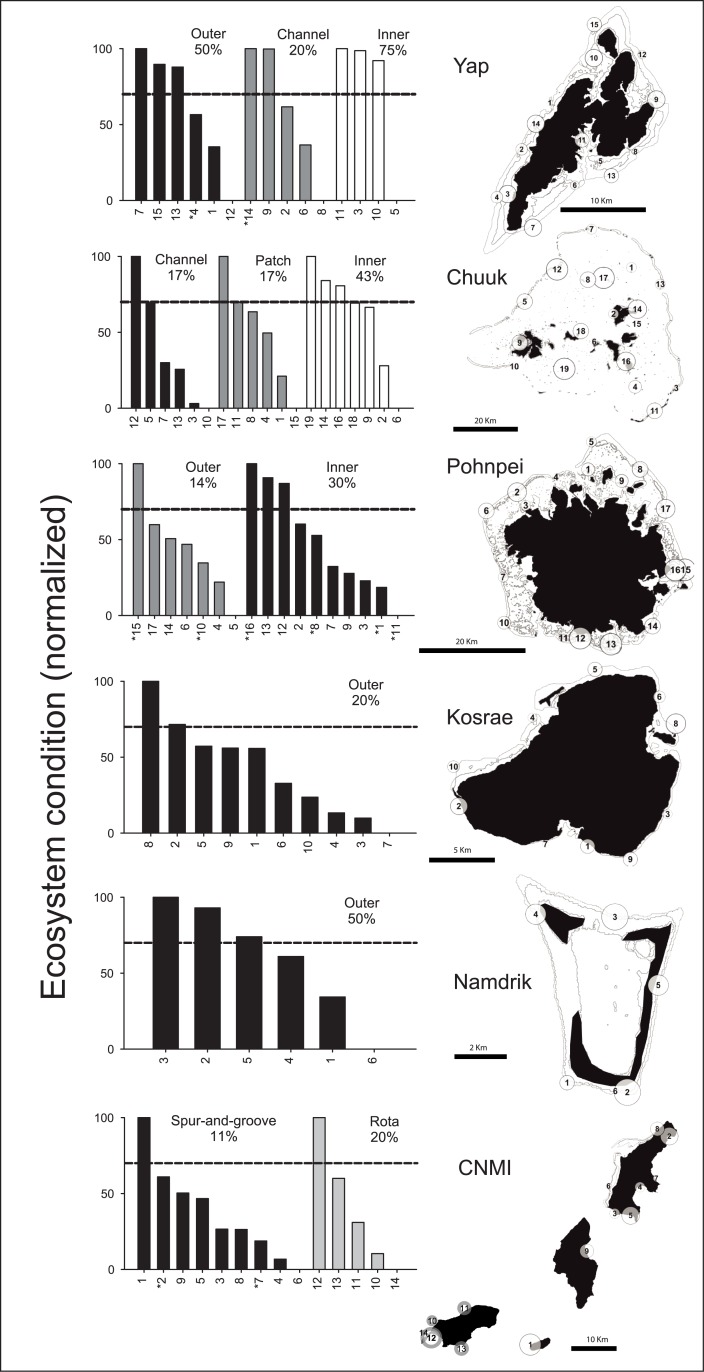
Normalized ecosystem-condition scores across Micronesia. Monitoring sites were stratified by reef habitat, management, geography, and/or wave exposure, as appropriate (S1 Fig). Dashed lines depict the ‘effective-conservation’ threshold used to assess progress towards the Micronesia-Challenge conservation goals. Percentages indicate the proportion of sites currently above the threshold. Site-circle sizes on the maps adjacent to the bar graphs were scaled by their normalized ecosystem-condition scores. Marine protected areas are designated on the bar graphs with an asterisk (*).

The percentage of reefs above the Micronesia-Challenge (MC) effective-conservation threshold was highest for Namdrik, Yap outer, and the inner reefs of Chuuk and Yap (43 to 75%). By contrast, the percentage of reefs that were above the effective-conservation threshold was lowest for the CNMI spur-and-groove reefs, Pohnpei outer reefs, and the patch and channel reefs of Chuuk (11 to 17%). Cumulatively, seven strata did not exceed the MC target of 30% (Fig 3). No-take marine protected areas (MPAs) varied greatly in their normalized ecosystem-condition scores, ranging from 15 to 100. These results suggested that there are clear differences in MPA effectiveness across Micronesia (Fig 3). The stability of ecosystem-condition scores was lowest for many of the same localities with low effective-conservation scores, including major reef habitats in Chuuk, CNMI, and Pohnpei noted above; yet there were some notable differences. For example, reefs in Rota (CNMI) had low ecosystem-condition scores coupled with low-to-moderate variances associated with these scores.

**Fig 4:**
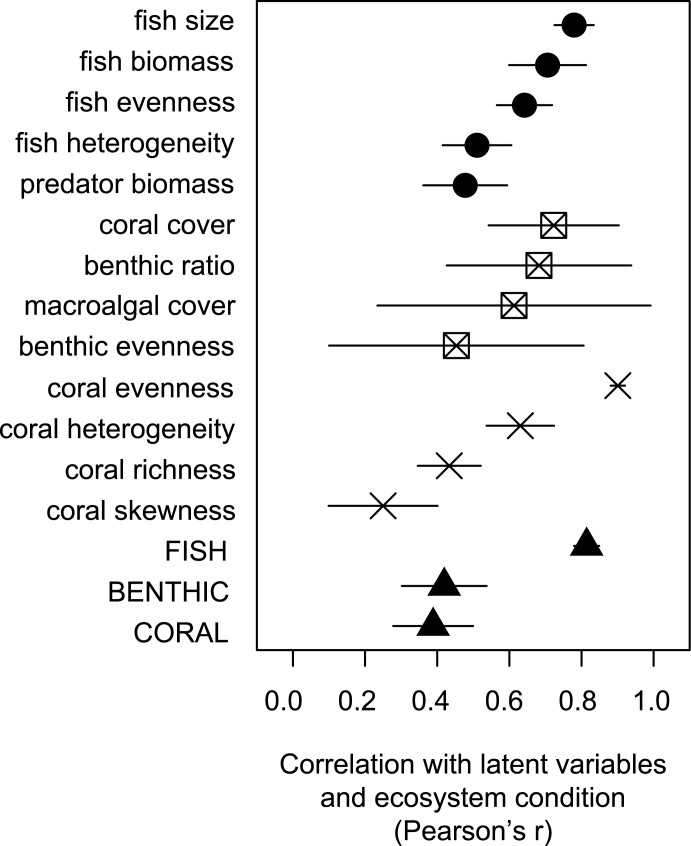
Sensitivity of ecosystem condition scores depicted by correlation coefficients between individual metrics and their respective latent variables (circles-fish, squares-benthic, and crosses-corals), or between latent variables and overall ecosystem condition (bottom triangles). Error bars (thin lines) represent standard deviations associated with the differing jurisdiction and reef-habitat strata.

Sensitivity analyses showed that fish size and biomass (excluding sharks), coral cover and benthic substrate ratios, and coral evenness and heterogeneity, respectively, had the strongest associations with the latent variables describing fish, benthic substrates, and corals (Pearson’s r > 0.7, Fig 4 and S3 Fig). These metrics also had the lowest site-level variation within major reef habitats (S2 Table). By contrast, predator biomass and fish-assemblage heterogeneity, benthic evenness, and the skewness and richness of coral assemblages provided the strongest independent contribution to the respective latent variables (Fig 4 and S3 Fig).

### Potential drivers of ecosystem condition

**Fig 5:**
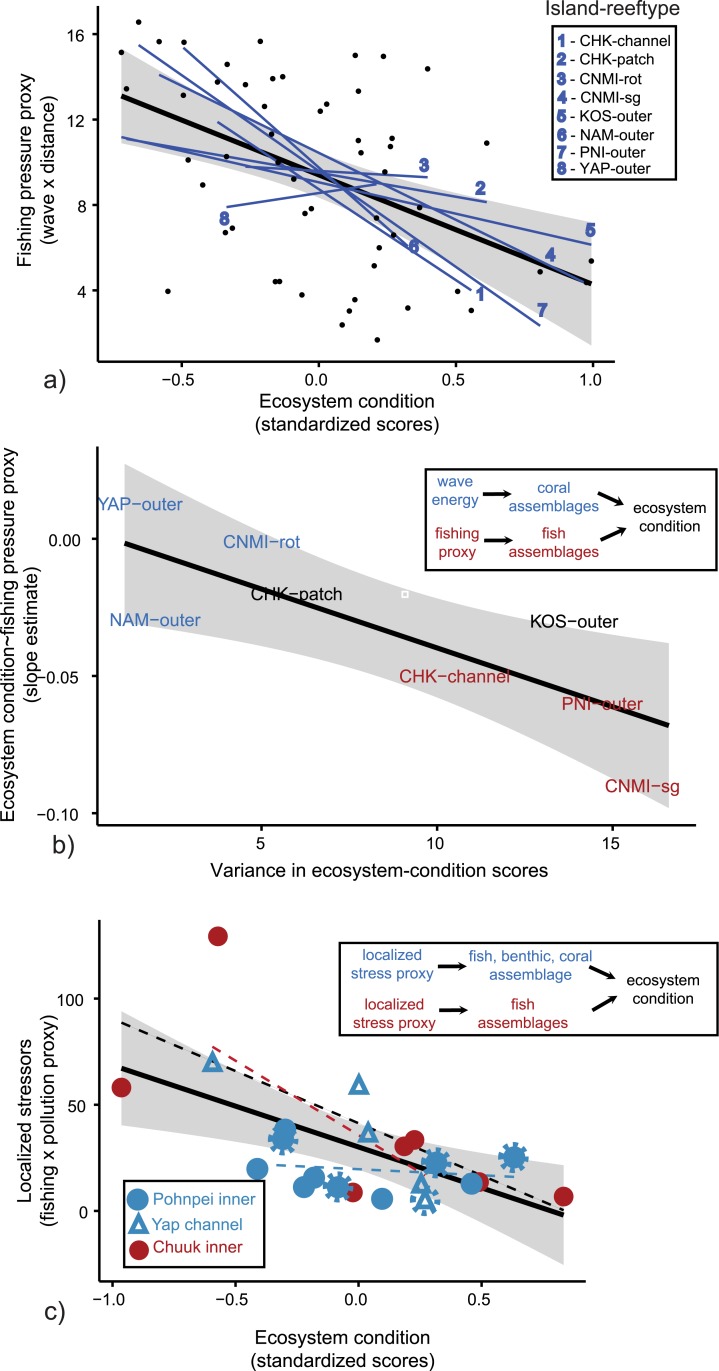
Regression models depicting the relationships between localized stressors and ecosystem condition. Fishing pressure predicted ecosystem condition on reefs > 5 km from land across Micronesia, with notable variation among islands and reef habitats (a). Independent slopes, in turn, predicted the variance among the top four non-normalized ecosystem condition scores (b, S2 Table), and also the relative influence of wave exposure—coral assemblages—ecosystem condition (blue), or the fishing pressure proxy-fish assemblages—ecosystem condition (red), or a direct relationship between the two integrated variables, fishing pressure proxy—ecosystem condition (black). Conversely, interactions between fishing and pollution proxies, termed localized stressors, best predicted condition for lagoon reefs <5 km from land (c), with stressors acting equally (blue) or unequally (red) across benthic, coral, and fish latent variables. Excluding marine protected areas (MPA, dashed circles around site symbols) did not improve the regional model fit, but removing two successful MPAs on Pohnpei (circles on furthest right) did improve the localized association between stressors and ecosystem condition.

While the normalized ecosystem-condition scores offered a means towards evaluating management at both local and regional scales, the gradient in scores provided the foundation for examining the influence of environmental regimes and potential stressors. Fishing pressure had the greatest effect on ecosystem condition across the outer reefs of Micronesia. Fishing pressure was evaluated by the interaction between wave energy and distance from the main fishing ports (Fig 5a). Mixed-effect models that allowed for random variation between the fishing proxy slopes and *y*-intercepts across strata did not improve model fit. In addition, the exclusion of marine protected areas did not significantly improve the model fit either. There was a weak, but highly-significant, association between fishing pressure and ecosystem condition (R^2^ = 0.20, P<0.001, df = 52), yet the magnitude of this relationship differed greatly across the region. Interestingly, independent slopes of these relationships were useful predictors of the variance in ecosystem condition within each stratum, defined here as the stability of effective-conservation scores that constituted progress towards MC goals (R^2^ = 0.61, P = 0.01, df = 6, Fig 5b). Thus, the magnitude of fishing pressure impacts should predict the likelihood of the reef status remaining stable through time. Furthermore, associations between the constituents of fishing proxies and ecosystem-condition scores revealed that the slopes of the model also predicted whether this effect was attributed to wave exposure alone (i.e., a natural environmental regime), distance from the main fishing ports, or both. Localities with large, negative slopes had the strongest covariance between the fishing proxies and the fish assemblages, and in turn had the strongest covariance between the fish assemblages and the ecosystem conditions (r-coefficients mean = 0.75 ± 0.03, Chuuk channel, Pohnpei outer, and CNMI spur-and-groove, red colors, Fig 5b). Conversely, the localities with small, negative slopes had weak covariances between distance from fishing access and fish latent variables in the case of Rota, CNMI (r = 0.45), and either positive or negative covariances between wave exposure and the latent variables describing coral assemblages that most influenced ecosystem condition scores. Depending upon the gradient of wave exposure around each island, a positive (e.g., Namdrik, 5.6°N 168°E, had low overall wave exposure but high wave exposure variance) or negative association (e.g., CNMI-Rota and Yap-outer, 14.1°N 145°E and 9.5°N 138°E, respectively, had high overall wave exposure and low exposure variance) existed with the coral assemblages. For Kosrae outer reefs and for Chuuk patch reefs no improved understanding resulted from examining individual constituents of the fishing proxy and ecosystem condition, as weak, negative slope existed between the two integrated variables.

The interaction between proxies for fishing and pollution had the strongest effect upon ecosystem condition across the inner lagoon reefs of Micronesia (Fig 5c). Generalized linear mixed-models did not improve the model fit beyond a global, linear model (R^2^ = 0.35, P = 0.002, df = 20). The exclusion of MPAs did not improve the explanatory power of localized stressors within regional models either (R^2^ = 0.32, P = 0.009, df = 15), but removal of two successful MPAs where the highest fish biomass existed did yield improved covariances between localized stressors and ecosystem condition for Pohnpei inner reefs (sites 16 and 12, Fig 3, S2 Table). Further examinations between the constituents of proxies to stressors and condition scores provided added details in one instance. For example, for the inner reefs of Chuuk, the interactive proxies to local stressors, fish assemblages, and ecosystem condition had the highest covariance structure (r-coefficients mean = 0.78 ± 0.2), positing that fish assemblages were both responsive to stressors and indicative of ecosystem condition. Elsewhere, the synergistic effects that were inferred through combined proxies and condition scores were strongest within both the Pohnpei and Yap lagoons.

## Discussion

Fishing pressure was a primary determinant of ecosystem condition across the majority (72%) of islands and reef habitats in Micronesia. High-wave exposure and far distances from major access ports were both beneficial to reef-fish populations and to overall ecosystem condition. Fishing pressure acted in combination with pollution to predict a declining ecosystem condition in the lagoons of Yap and Pohnpei, where poor land-use had a more context-dependent role that was most pronounced in the few, urbanized watersheds adjacent to inner reefs of high islands. While localized stressors clearly had a consistent, regional influence, the magnitude of this influence differed substantially. Reef habitats that were most impacted by localized stressors (i.e., greatest slopes, Fig 5) also had the least stable ecosystem-condition scores. For these localities, progress towards the Micronesia Challenge conservation goals was more dependent upon site-based characteristics, such as high-wave exposure, far distances from human populations, and effective marine protected areas (MPAs). Yet, placing a greater dependency on inconsistent or intermittent regimes threatens resource sustainability through time [42]. Furthermore, MPAs had a limited efficacy when considered collectively as a regional management regime, despite the fact that localized MPA success stories are supported in one instance on Yap [43], and in two instances on Pohnpei. Elsewhere, reef habitats with the weakest slopes had more stable ecosystem condition scores, and a greater influence of natural environmental regimes in driving these scores on Namdrik, Rota, and Yap outer reefs.

While spatially-extensive fisheries polices exist across Micronesia, they are rare (S3 Table), and not well aligned with the most sensitive attributes of fish assemblages reported here: fish- size, heterogeneity, and predator biomass. Because the observed fish assemblages were disproportionally weighted by herbivores (57% of the biomass, and 72% of the population densities were herbivores), these findings suggest that grazing rates and herbivore-functional diversity were (i) most sensitive to fishing pressure, and (ii) indicative of fundamental trophic interactions driven by fish assemblages on coral reefs [20,29,44]. We add that predator presence served to enhance both of these attributes. Predator biomass predicted the heterogeneity of herbivores across our study islands, whether examining data at the island-level or across sites within 5 of the 8 study islands. These results resonate with studies showing that predators act as couplers to fast and slow-energy channels within food webs and facilitate the stable persistence of species with differing growth strategies [26,28]. Elsewhere, in remote atolls across the Pacific Ocean, reduced apex-predator biomass has predicted low functional diversity among herbivore guilds [37,45], with consequential increases in macroalgae and the survival of fewer, stress-tolerant corals. Despite these lines of evidence, comprehensive policies regulating night-time spearfishing, exports, size-at-capture, and catch quotas are lacking in many instances (S3 Table), while night-time spearfishing contributes disproportionally to market landings across Micronesia [4] and vulnerabity to fishing pressure is reflected by size-and-species-based life-history traits [46]. We suggest that the Micronesia Challenge represents a novel management landscape from which common, improved policies might emerge and focus upon these sensitive metrics.

Predicting the impacts of pollution on benthic and coral assemblages is well documented, but predicting the impacts of pollution on reef-fish assemblages remains a priority for understanding ecosystem dynamics in Micronesia’s lagoons. Growing evidence of sediment impacts to reef-fish assemblages include: (i) reduced juvenile recruitment and settlement success because of sedimentation, (ii) altered adult movement patterns, (iii) a disruption of physiological processes such as reproduction and respiration, and (iv) habitat destruction from the loss of coral [47]. For instance, water visibility consistently below 10 m on the inshore Great Barrier Reef was associated with reduced herbivore richness and abundance [48]. However, it remains unclear what influence moderate levels of pollution have on the overall abundance of food fishes and the functional roles of trophic guilds. Cascading responses to enhanced bottom-up production are predicted to yield a unimodal relationship, improving responsive food-fish populations at first, then facilitating unstable population dynamics as food resources become less limiting, and inter-species competition increases [49,50]. Thus, major fish guilds may be indirectly impacted from poor water quality through competition and directly by physiological tolerances, but causative evidence remains limited, especially across moderate ranges of pollution.

### Summary and conclusions

A growing number of studies continue to examine coral reefs across large spatial scales and highlight factors that have contributed most to the observed ecological dynamics [51–55]. We conclude that teasing apart the impacts of key abiotic stressors (i.e., non-biological measures of fishing pressure predict reduced fish sizes) from the biological associations (i.e., more herbivores predict reduced macroalgae) can best catalyze the feedback that is required between science and management. Because ecosystem-condition scores were influenced by both environmental gradients and localized stressors, partitioning the variance structure was necessary before properly evaluating Micronesia-Challenge (MC) conservation targets. In other words, reef habitats will vary considerably in their response to local management. Less effort will be required to attain conservation targets in habitats where high-wave exposure, or far distances from urban centers exist. Whereas habitats close to urban centers may require more management effort and may show less of a positive response to management than distant sites. We conclude that fish assemblages appeared to have a hierarchical influence upon coral-reef ecosystems in Micronesia compared with localized pollution. Prioritizing management upon herbivore size and diversity, which are both mediated by predators, is expected to best preserve the underlying trophic relationships responsible for the ecosystem services that coral reefs provide to Micronesian societies in the face of ongoing climate change.

## Supporting Information

S1 Fig Examples of pressing questions driving monitoring designs for collaborative coral-reef monitoring across Micronesia, and their spatial and temporal scales of operation. (JPG)

S2 Fig Principle components ordination plots of benthic substrate abundances across jurisdiction-reef types in Micronesia. Dashed lines indicate significant differences based upon permutational multivariate analyses of comparisons. Influential substrate categories are shown on the plots, with their location depicting their affinity with major reef types. Locations of substrates categories on the plots were derived from spearman rank correlation coefficients with PCO axes (ρ > 0.5).
(JPG)

S3 Fig Correlation matrices between individual metrics within their corresponding latent variable groupings: fish (a), coral (b), and benthic substrates (c). Colors indicate significant correlation (P<0.05, blue-positive, red-negative), with narrower ellipses and darker colors indicating stronger associations.
(JPG)

S1 Table Biological metrics used to evaluate coral-reef ecosystem conditions that were related to reef processes and ecological principles. (PDF)

S2 Table Site-based characteristics and biological metrics used for data analyses. (PDF)

S3 Table Summary of regulations for coral-reef fisheries and watershed pollution in each Micronesian jurisdiction. (PDF)
