# Supplementary figures and images for: The Micronesia Challenge: Assessing the Relative Contribution of Stressors on Coral Reefs to Facilitate Science-to-Management Feedback
Source: PLoS One. 2015 Jun 18;10(6):e0130823. doi: 10.1371/journal.pone.0130823 (PMC4473011; doi:10.1371/journal.pone.0130823)

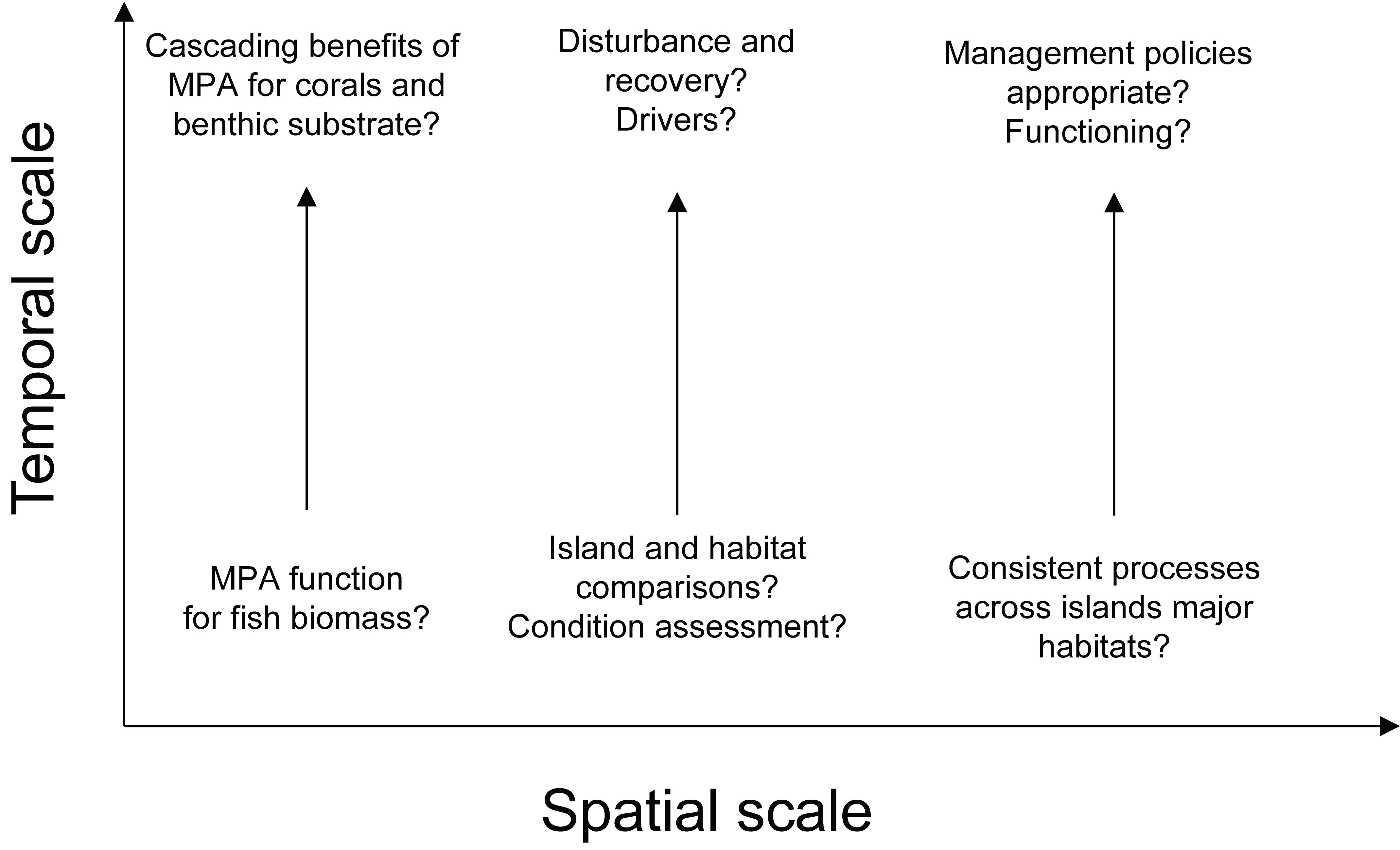

Supplement: S1 Fig — (JPG) [file pone.0130823.s001.jpg]

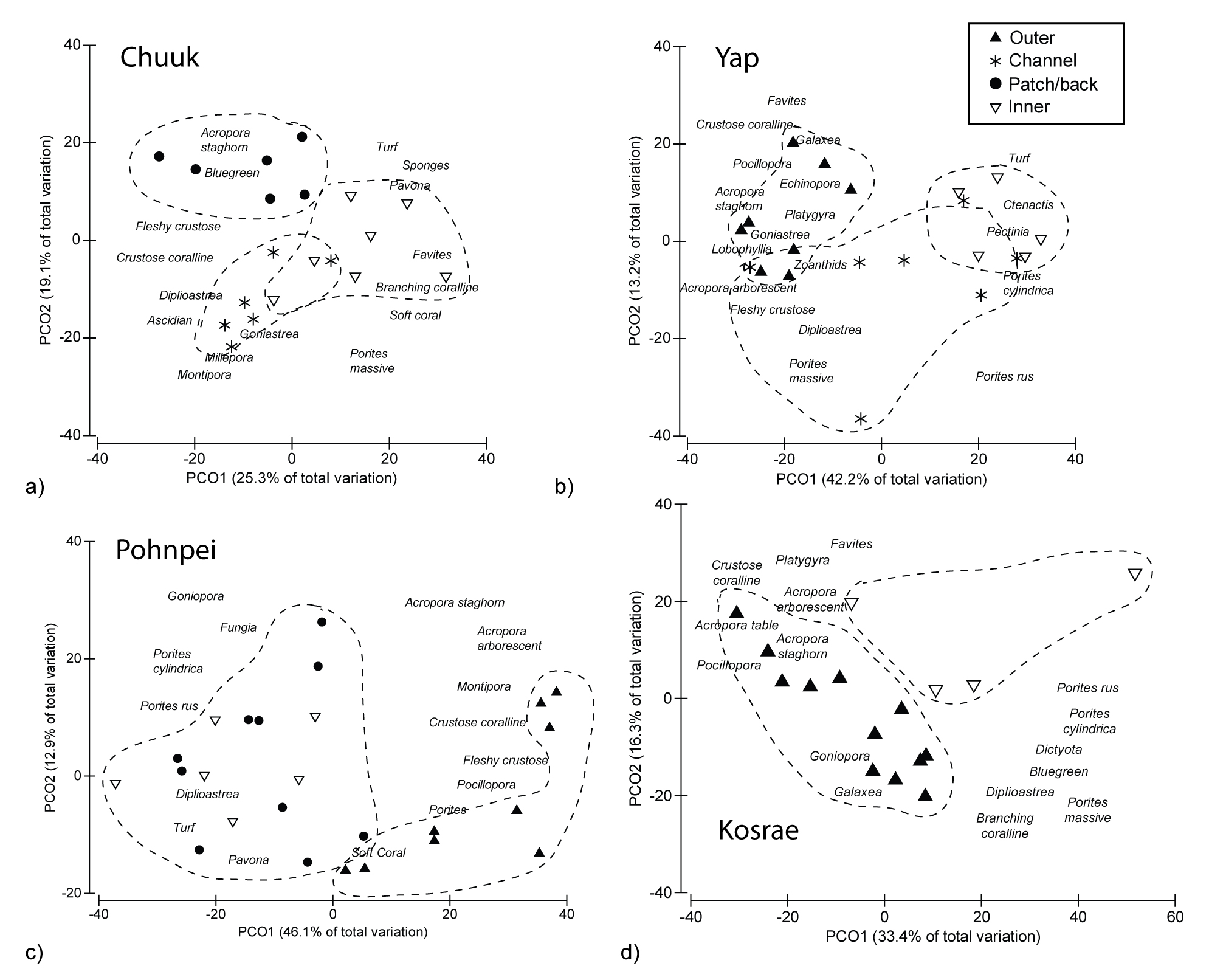

Supplement: S2 Fig — Dashed lines indicate significant differences based upon permutational multivariate analyses of comparisons. Influential substrate categories are shown on the plots, with their location depicting their affinity with major reef types. Locations of substrates categories on the plots were derived from spearman rank correlation coefficients with PCO axes (ρ > 0.5). (JPG) [file pone.0130823.s002.jpg]

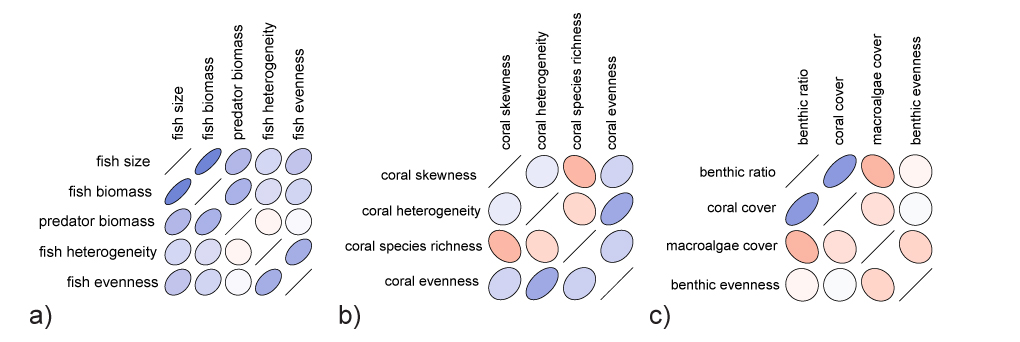

Supplement: S3 Fig — Colors indicate significant correlation (P<0.05, blue-positive, red-negative), with narrower ellipses and darker colors indicating stronger associations. (JPG) [file pone.0130823.s003.jpg]
