# Supplementary material for: The Micronesia Challenge: Assessing the Relative Contribution of Stressors on Coral Reefs to Facilitate Science-to-Management Feedback
Source: PLoS One. 2015 Jun 18;10(6):e0130823. doi: 10.1371/journal.pone.0130823 (PMC4473011; doi:10.1371/journal.pone.0130823)
Supplement: S1 Table — (PDF) [file pone.0130823.s004.pdf]

Table S1. Biological metrics used to evaluate coral-reef ecosystem conditions that were related reef processes and ecological principles.

METRIC	KEY PROCESSES	FOUNDATIONAL PRINCIPLES	SUPPORTING CITATIONS
**FISH ASSEMBLAGE SIZE**	Grazing, disturbance resistance, recovery potential, distribution of diversity across trophic guilds, sustainable provisioning	Allometry, reproduction, niche partitioning, distribution of biomass across fast/slow energy pathways in food webs, size-based fishery regulations	[1] [2] [3] [4] [5] [6] [7] [8] [9] [10,11]
**FISH ASSEMBLAGE BIOMASS**	Disturbance resistance, recovery potential, carbon sequestration, sustainable provisioning	Food web stability, Nutrient cycling, harvesting quotas,	[11-17]
**PREDATOR BIOMASS**	Competitive dominance in lower trophic guilds, carbon sequestration, ecosystem function	Predation, competition, diversity maintenance, trophic cascades, food web stability	[13,14,18-20]
**FISH ASSEMBLAGE HETEROGENEITY AND EVENNESS**	Disturbance resistance, recovery potential	Response diversity, functional redundancy, food web stability	[18,21-27]
**BENTHIC SUBSTRATE RATIO[1]**	Reef calcification and accretion, carbon sequestration	Competition, slow energy pathways in food webs	[10,16,28-30]
**CORAL COVER**	Habitat formation and complexity, reef calcification and accretion, carbon sequestration	Nutrient cycling, fundamental niche creation, slow energy pathways in food webs	[31-34]
**MACROALGAL COVER**	Competition, grazing, carbon recycling	Nutrient cycling, herbivore response, fast energy pathways in food webs	[16,28-30]
**CORAL EVENNESS, SPECIES RICHNESS, AND ASSEMBLAGE HETEROGENEITY**	Habitat complexity, disturbance resistance, recovery potential	Response diversity, functional diversity, food web stability	[4, 31-35]
**CORAL ASSEMBLAGE SKEWNESS**	Recruitment, habitat complexity, habitat formation	Allometry, fundamental niche creation, fast/slow energy pathways in food	[16,33,36,37]

		webs, population dynamics	

References

1. Birkeland C, Dayton PK (2005) The importance in fishery management of leaving the big ones. Trends Ecol Evol 20: 356-358.
2. Lokrantz J, Nyström M, Thyresson M, Johansson C (2008) The non-linear relationship between body size and function in parrotfishes. Coral Reefs 27: 967-974.
3. Sweatman H (2008) No-take reserves protect coral reefs from predatory starfish. Curr Biol 18: R598-R599.
4. Houk P, Benavente D, Fread V (2012) Characterization and evaluation of coral reefs around Yap Proper, Federated States of Micronesia. Biodiver Conserv 21: 2045-2059.
5. Piet G, Pet J, Guruge W, Vijverberg J, Van Densen W (1999) Resource partitioning along three niche dimensions in a size-structured tropical fish assemblage. Canadian J Fish Aquat Sci 56: 1241-1254.
6. Welsh J, Goatley C, Bellwood D (2013) The ontogeny of home ranges: evidence from coral reef fishes. Proc Roy Soc B: Biol Sci 280: 20132066.
7. Hutchinson GE (1959) Homage to Santa Rosalia or why are there so many kinds of animals? Am Nat: 145-159.
8. Worm B, Hilborn R, Baum JK, Branch TA, Collie JS, et al. (2009) Rebuilding global fisheries. Science 325: 578-585.
9. Rooney N, McCann KS, Moore JC (2008) A landscape theory for food web architecture. Ecol Lett 11: 867-881.
10. Rooney N, McCann KS (2012) Integrating food web diversity, structure and stability. Trends Ecol Evol 27: 40-46.
11. Banse K (2007) Do we live in a largely top-down regulated world ? J Biosci 32: 791-796.
12. Vanni MJ (2002) Nutrient cycling by animals in freshwater ecosystems. Annu Rev Ecol System: 341-370.
13. Estes JA, Terborgh J, Brashares JS, Power ME, Berger J, et al. (2011) Trophic downgrading of planet Earth. Science 333: 301-306.
14. Vanni MJ, Layne CD, Arnott SE (1997) “Top-down” trophic interactions in lakes: effects of fish on nutrient dynamics. Ecology 78: 1-20.
15. Costello C, Gaines SD, Lynham J (2008) Can catch shares prevent fisheries collapse? Science 321: 1678-1681.
16. Hoegh-Guldberg O, Mumby P, Hooten A, Steneck R, Greenfield P, et al. (2007) Coral reefs under rapid climate change and ocean acidification. Science 318: 1737-1742.
17. Mumby PJ, Dahlgren CP, Harborne AR, Kappel CV, Micheli F, et al. (2006) Fishing, trophic cascades, and the process of grazing on coral reefs. Science 311: 98-101.
18. Houk P, Benavente D, Iguel J, Johnson S, Okano R (201r) Coral reef disturbance and recovery dynamics differ across gradients of localized stressors in the Mariana Islands. PLoS ONE 9:e105731.
19. Burkepile DE, Allgeier JE, Shantz AA, Pritchard CE, Lemoine NP, et al. (2013) Nutrient supply from fishes facilitates macroalgae and suppresses corals in a Caribbean coral reef ecosystem. Scientific Reports 3: 1493.
20. Bascompte J, Melián CJ (2005) Simple trophic modules for complex food webs. Ecology 86: 2868-2873.

21. Mellin C, Bradshaw C, Fordham D, Caley M (2014) Strong but opposing β-diversity–stability relationships in coral reef fish communities. Proc Roy Soc B: Biol Sci 281: 20131993.
22. Thibaut LM, Connolly SR, Sweatman HP (2012) Diversity and stability of herbivorous fishes on coral reefs. Ecology 93: 891-901.
23. Allesina S, Tang S (2012) Stability criteria for complex ecosystems. Nature 483: 205-208.
24. Myers RA, Worm B (2003) Rapid worldwide depletion of predatory fish communities. Nature 423: 280-283.
25. Bellwood DR (2003) Origins and escalation of herbivory in fishes: a functional perspective. Paleobiology 29: 71-83.
26. Rasher DB, Hoey AS, Hay ME (2013) Consumer diversity interacts with prey defenses to drive ecosystem function. Ecology 94: 1347-1358.
27. Burkepile DE, Hay ME (2008) Herbivore species richness and feeding complementarity affect community structure and function on a coral reef. Proc Nat Acad Sci 105: 16201-16206.
28. Lirman D (2001) Competition between macroalgae and corals: effects of herbivore exclusion and increased algal biomass on coral survivorship and growth. Coral Reefs 19: 392-399.
29. Tanner JE (1995) Competition between scleractinian corals and macroalgae: an experimental investigation of coral growth, survival and reproduction. J Exp Mar Biol Ecol 190: 151-168.
30. Moore JC, Berlow EL, Coleman DC, Ruiter PC, Dong Q, et al. (2004) Detritus, trophic dynamics and biodiversity. Ecol Lett 7: 584-600.
31. Graham NA, Wilson SK, Jennings S, Polunin NV, Bijoux JP, et al. (2006) Dynamic fragility of oceanic coral reef ecosystems. Proc Nat Acad Sci 103: 8425-8429.
32. Alvarez-Filip L, Dulvy NK, Gill JA, Côté IM, Watkinson AR (2009) Flattening of Caribbean coral reefs: region-wide declines in architectural complexity. Proc Roy Soc B: Biol Sci: rspb20090339.
33. Almany GR (2004) Differential effects of habitat complexity, predators and competitors on abundance of juvenile and adult coral reef fishes. Oecologia 141: 105-113.
34. Wilson S, Fisher R, Pratchett M, Graham N, Dulvy N, et al. (2008) Exploitation and habitat degradation as agents of change within coral reef fish communities. Glob Change Biol 14: 2796-2809.
35. Baskett ML, Fabina NS, Gross K (2014) Response Diversity Can Increase Ecological Resilience to Disturbance in Coral Reefs. Am Nat 184: E16-E31.
36. Porter JW (1976) Autotrophy, heterotrophy, and resource partitioning in Caribbean reef-building corals. Am Nat: 731-742.
37. Meesters E, Hilterman M, Kardinaal E, Keetman M, De Vries M, et al. (2001) Colony size-frequency distributions of scleractinian coral populations: spatial and interspecific variation. Mar Ecol Prog Ser 209: 43-54.
