# Supplementary material for: The Micronesia Challenge: Assessing the Relative Contribution of Stressors on Coral Reefs to Facilitate Science-to-Management Feedback
Source: PLoS One. 2015 Jun 18;10(6):e0130823. doi: 10.1371/journal.pone.0130823 (PMC4473011; doi:10.1371/journal.pone.0130823)
Supplement: S2 Table — (PDF) [file pone.0130823.s005.pdf]

Table S2. Site-based characteristics and biological metrics used for data analyses.

| Jurisdiction | Island | Site | Local name | GPS X | GPS Y | Reef type | MPA Status | Wave Exposure (J/m3) | Distance from major fishing port (km) | Fishing proxy (standardized by strata and combined) | Disturbed land in watershed (km2) | Distance from discharge (km) | Pollution proxy (standardized by strata and combined) |
|---|---|---|---|---|---|---|---|---|---|---|---|---|---|
| Commonwealth of the Northern Mariana Islands | Aguijan | CNMI-1 | AGU_12 | 342807 | 1642480 | Spur-and-groove | No | 629.8 | 38.6 | 4.37 | 0.04 | 0.20 | 2.58 |
| Commonwealth of the Northern Mariana Islands | Rota | CNMI-10 | ROT-6_12 | 300459 | 1566145 | Rota raised Holocene | No | 365.4 | 3.4 | 12.61 | 0.19 | 0.18 | 4.01 |
| Commonwealth of the Northern Mariana Islands | Rota | CNMI-11 | RR_12 | 308740 | 1570072 | Rota raised Holocene | No | 965.3 | 12.8 | 3.79 | 0.29 | 0.12 | 6.51 |
| Commonwealth of the Northern Mariana Islands | Rota | CNMI-12 | SAHY_12 | 299569 | 1563743 | Rota raised Holocene | No | 161.1 | 0.5 | 14.37 | 0.11 | 0.18 | 3.03 |
| Commonwealth of the Northern Mariana Islands | Rota | CNMI-13 | TALK_12 | 305271 | 1561044 | Rota raised Holocene | No | 584.0 | 7.2 | 3.56 | 0.56 | 0.22 | 7.21 |
| Commonwealth of the Northern Mariana Islands | Rota | CNMI-14 | WHB_12 | 298271 | 1563772 | Rota raised Holocene | No | 407.0 | 0.6 | 13.63 | 0.09 | 0.39 | 0.38 |
| Commonwealth of the Northern Mariana Islands | Saipan | CNMI-2 | BI_12 | 372724 | 1687351 | Spur-and-groove | Yes | 501.2 | 25.6 | 7.88 | 0.09 | 0.21 | 1.47 |
| Commonwealth of the Northern Mariana Islands | Saipan | CNMI-3 | COP_12 | 360977 | 1670710 | Spur-and-groove | No | 128.7 | 7.42 | 13.91 | 0.29 | 0.19 | 5.47 |
| Commonwealth of the Northern Mariana Islands | Saipan | CNMI-4 | LLB#1_12 | 366425 | 1676402 | Spur-and-groove | No | 224.3 | 21.4 | 10.11 | 0.09 | 0.18 | 5.48 |
| Commonwealth of the Northern Mariana Islands | Saipan | CNMI-5 | OBY_12_2 | 364397 | 1670287 | Spur-and-groove | No | 127.8 | 10.9 | 13.33 | 0.13 | 0.18 | 5.85 |
| Commonwealth of the Northern Mariana Islands | Saipan | CNMI-6 | OGND_12 | 359633 | 1676634 | Spur-and-groove | No | 304.0 | 1.7 | 15.65 | N/A | N/A | N/A |
| Commonwealth of the Northern Mariana Islands | Saipan | CNMI-7 | TANK_12 | 369943 | 1678305 | Spur-and-groove | Yes | 2530.1 | 27.4 | 1.86 | 1.16 | 0.20 | 7.63 |
| Commonwealth of the Northern Mariana Islands | Saipan | CNMI-8 | WB_11 | 370155 | 1688934 | Spur-and-groove | No | 360.5 | 15.2 | 11.31 | N/A | N/A | N/A |
| Commonwealth of the Northern Mariana Islands | Tinian | CNMI-9 | TIN - 1 (12) | 355222 | 1662018 | Spur-and-groove | No | 2215.9 | 14.8 | 5.15 | 0.04 | 0.21 | 1.37 |
| Federated States of Micronesia | Chuuk | CHK-1 | Puwe | 837023 | 377259 | Patch | No | 247.0 | 12.6 | 8.94 | N/A | N/A | N/A |
| Federated States of Micronesia | Chuuk | CHK-10 | Fourupw | 806821 | 341489 | Channel | No | 262.3 | 37.4 | 13.76 | N/A | N/A | N/A |
| Federated States of Micronesia | Chuuk | CHK-11 | Wininen | 793255 | 384328 | Patch | No | 426.5 | 36.2 | 1.68 | N/A | N/A | N/A |
| Federated States of Micronesia | Chuuk | CHK-12 | Oun | 836404 | 354517 | Channel | No | 2021.6 | 21.6 | 3.06 | N/A | N/A | N/A |
| Federated States of Micronesia | Chuuk | CHK-13 | Moch | 831950 | 386042 | Channel | No | 195.6 | 13.8 | 14.00 | N/A | N/A | N/A |
| Federated States of Micronesia | Chuuk | CHK-14 | Sapuk | 824304 | 379043 | Inner | No | 40.0 | 7.8 | 12.50 | 0.04 | 0.58 | 2.67 |
| Federated States of Micronesia | Chuuk | CHK-15 | Apichun | 819913 | 378854 | Patch | No | 59.9 | 12.2 | 13.44 | N/A | N/A | N/A |
| Federated States of Micronesia | Chuuk | CHK-16 | Manukun | 808363 | 375577 | Inner | No | 117.9 | 18.2 | 7.06 | 0.04 | 0.29 | 4.31 |
| Federated States of Micronesia | Chuuk | CHK-17 | Fanangal | 833887 | 368737 | Patch | No | 195.0 | 9.9 | 10.90 | N/A | N/A | N/A |
| Federated States of Micronesia | Chuuk | CHK-18 | Och | 817621 | 361795 | Inner | No | 318.8 | 13.1 | 6.62 | 0.01 | 0.81 | 1.31 |
| Federated States of Micronesia | Chuuk | CHK-19 | none yet | 806077 | 356745 | Inner | No | 665.1 | 24.7 | 1.56 | N/A | N/A | 4.34 |
| Federated States of Micronesia | Chuuk | CHK-2 | Truk Stop | 822864 | 371782 | Inner | No | 229.7 | 2.9 | 12.20 | 0.71 | 0.33 | 10.60 |
| Federated States of Micronesia | Chuuk | CHK-3 | Sanat | 800230 | 390961 | Channel | No | 157.3 | 32.3 | 6.71 | N/A | N/A | N/A |
| Federated States of Micronesia | Chuuk | CHK-4 | Aroch | 800578 | 378381 | Patch | No | 144.5 | 28.5 | 7.60 | N/A | N/A | N/A |
| Federated States of Micronesia | Chuuk | CHK-5 | Oranu | 826638 | 344641 | Channel | No | 136.7 | 28.7 | 6.59 | N/A | N/A | N/A |
| Federated States of Micronesia | Chuuk | CHK-6 | Parem | 814354 | 365876 | Inner | No | 11.4 | 11.2 | 11.30 | 0.07 | 0.20 | 5.14 |
| Federated States of Micronesia | Chuuk | CHK-7 | Piis | 848755 | 365095 | Channel | No | 228.0 | 25.3 | 9.21 | N/A | N/A | N/A |
| Federated States of Micronesia | Chuuk | CHK-8 | Falos | 833313 | 363960 | Patch | No | 0.2 | 11.8 | 15.00 | N/A | N/A | N/A |
| Federated States of Micronesia | Chuuk | CHK-9 | Nematon | 814047 | 343125 | Inner | No | 2.7 | 40.2 | 15.54 | 0.07 | 0.92 | 0.87 |
| Federated States of Micronesia | Kosrae | KOS-1 | Buoy-16 | 582358 | 276178 | Outer | No | 127.5 | 2.6 | 14.96 | 0.11 | 0.25 | 5.06 |
| Federated States of Micronesia | Kosrae | KOS-3 | Buoy-8 | 584519 | 281554 | Outer | No | 2382.2 | 6.9 | 3.96 | 0.20 | 1.27 | 1.73 |
| Federated States of Micronesia | Kosrae | KOS-4 | Buoy-TS | 591012 | 272464 | Outer | No | 122.0 | 2.1 | 15.62 | 0.47 | 1.49 | 1.12 |
| Federated States of Micronesia | Kosrae | KOS-5 | Buoy-44 | 553424 | 167286 | Outer | No | 813.0 | 4.1 | 10.73 | 0.36 | 0.63 | 7.12 |
| Federated States of Micronesia | Kosrae | KOS-6 | Buoy-50 | 553002 | 278247 | Outer | No | 2372.3 | 6.1 | 4.41 | 0.09 | 0.35 | 4.33 |
| Federated States of Micronesia | Kosrae | KOS-7 | Buoy-20 | 582503 | 273344 | Outer | No | 239.8 | 2.1 | 15.14 | 0.04 | 0.65 | 2.63 |
| Federated States of Micronesia | Kosrae | KOS-2 | Buoy-27 | 585054 | 267521 | Outer | No | 156.8 | 11.2 | 3.95 | 0.01 | 0.68 | 2.18 |
| Federated States of Micronesia | Kosrae | KOS-8 | Buoy-53 | 590883 | 281512 | Outer | No | 2388.2 | 4.2 | 5.38 | N/A | N/A | N/A |
| Federated States of Micronesia | Kosrae | KOS-9 | Buoy-14 | 581642 | 277193 | Outer | No | 788.6 | 5.3 | 9.55 | 0.19 | 0.20 | 6.85 |
| Federated States of Micronesia | Kosrae | KOS-10 | Buoy-31 | 587580 | 267346 | Outer | No | 200.4 | 8.8 | 6.91 | 0.01 | 0.98 | 1.48 |
| Federated States of Micronesia | Pohnpei | PNI-1 | Sapwitik MPA Inner Reef | 774869 | 414498 | Inner | Yes | 33.6 | 5.74 | 7.44 | 4.64 | 5.16 | 4.55 |
| Federated States of Micronesia | Pohnpei | PNI-9 | Pearem patch reef | 773142 | 419563 | Inner | No | 39.3 | 12.9 | 5.20 | 0.18 | 3.55 | 2.16 |
| Federated States of Micronesia | Pohnpei | PNI-10 | Black Coral | 751750 | 401833 | Outer | Yes | 151.4 | 31.1 | 9.99 | N/A | N/A | N/A |
| Federated States of Micronesia | Pohnpei | PNI-11 | Laiap Island | 749443 | 410766 | Inner | Yes | 6.8 | 37.8 | 7.21 | 0.30 | 2.74 | 2.74 |
| Federated States of Micronesia | Pohnpei | PNI-2 | Palikir Channel | 771437 | 403790 | Inner | No | 23.9 | 11.2 | 9.24 | 0.71 | 6.84 | 0.62 |
| Federated States of Micronesia | Pohnpei | PNI-12 | Nahtik Inner | 749411 | 413314 | Inner | No | 2.2 | 40.9 | 7.03 | 0.39 | 2.12 | 3.23 |
| Federated States of Micronesia | Pohnpei | PNI-13 | Nan Mwokil | 748516 | 417939 | Inner | No | 0.3 | 45.3 | 6.07 | 0.43 | 4.14 | 2.11 |
| Federated States of Micronesia | Pohnpei | PNI-14 | Nan Pwin | 751234 | 424215 | Outer | No | 2403.0 | 53.1 | 2.38 | 0.35 | 2.48 | N/A |
| Federated States of Micronesia | Pohnpei | PNI-15 | Nan Wap Outer | 759729 | 429066 | Outer | Yes | 2367.0 | 32.9 | 4.87 | 2.78 | 7.63 | N/A |
| Federated States of Micronesia | Pohnpei | PNI-16 | Nan Wap Inner | 759707 | 427638 | Inner | Yes | 2.8 | 35.7 | 8.28 | 2.78 | 5.30 | 3.04 |
| Federated States of Micronesia | Pohnpei | PNI-17 | Madamken | 768932 | 426178 | Outer | No | 2367.9 | 23.6 | 6.00 | N/A | N/A | N/A |
| Federated States of Micronesia | Pohnpei | PNI-3 | Palikir Inner Reef (Mwahng) | 769443 | 405093 | Inner | No | 5.2 | 11.7 | 13.85 | 0.07 | 2.03 | 2.76 |
| Federated States of Micronesia | Pohnpei | PNI-4 | Main Channel (Sokehs) | 773711 | 409658 | Outer | No | 24.4 | 5.4 | 16.56 | 4.59 | 5.23 | N/A |
| Federated States of Micronesia | Pohnpei | PNI-5 | Nan Mweli Outer Reef | 779036 | 414985 | Outer | No | 288.1 | 9.9 | 14.58 | N/A | N/A | N/A |
| Federated States of Micronesia | Pohnpei | PNI-6 | Pwukihn Dawahk Outer Reef | 768618 | 399166 | Outer | No | 220.5 | 18.8 | 12.72 | N/A | N/A | N/A |
| Federated States of Micronesia | Pohnpei | PNI-7 | Pehleng Channel | 758640 | 401697 | Inner | No | 29.9 | 24.1 | 5.97 | 2.52 | 5.51 | 2.62 |
| Federated States of Micronesia | Pohnpei | PNI-8 | Mwahnd MPA Inner Reef | 774875 | 422339 | Inner | Yes | 1.7 | 14.1 | 14.10 | 0.18 | 6.23 | 0.79 |
| Federated States of Micronesia | Yap | YAP-1 | Atliw | 1058744 | 181245 | Outer | No | 839.5 | 19.9 | 10.26 | 0.14 | 1.23 | N/A |
| Federated States of Micronesia | Yap | YAP-9 | Gafnuw Channel | 1059013 | 192630 | Channel | No | 202.4 | 19.6 | 5.30 | 1.61 | 3.20 | 1.77 |
| Federated States of Micronesia | Yap | YAP-10 | M'il Inner Reef | 1063528 | 185988 | Inner | No | 19.0 | 11.4 | 4.77 | 0.19 | 1.56 | 2.10 |
| Federated States of Micronesia | Yap | YAP-11 | Pakel | 1054712 | 184646 | Inner | No | 1.1 | 3.1 | 14.10 | 1.32 | 1.67 | 3.60 |

| Jurisdiction | Island | Site | Local name | GPS X | GPS Y | Reef type | MPA Status | Wave Exposure (J/m3) | Distance from major fishing port (km) | Fishing proxy (standardized by strata and combined) | Disturbed land in watershed (km2) | Distance from discharge (km) | Pollution proxy (standardized by strata and combined) |
|---|---|---|---|---|---|---|---|---|---|---|---|---|---|
| Federated States of Micronesia | Yap | YAP-12 | Toruw | 1063860 | 191153 | Outer | No | 1772.1 | 22.6 | 4.42 | N/A | N/A | N/A |
| Federated States of Micronesia | Yap | YAP-14 | Nimpal Channel | 1056509 | 179732 | Channel | Yes | 243.2 | 21.2 | 4.09 | 0.18 | 0.94 | 1.21 |
| Federated States of Micronesia | Yap | YAP-2 | Gachug | 1053766 | 178195 | Channel | No | 6.6 | 23.7 | 7.48 | 0.98 | 1.39 | 4.86 |
| Federated States of Micronesia | Yap | YAP-3 | N'ef Blue Hole | 1048969 | 176663 | Inner | No | 8.1 | 26.1 | 4.69 | 0.02 | 0.72 | 4.06 |
| Federated States of Micronesia | Yap | YAP-4 | Reey Outer Reef | 1048630 | 175506 | Outer | Yes | 808.8 | 24.1 | 7.83 | N/A | N/A | N/A |
| Federated States of Micronesia | Yap | YAP-5 | Af Blue Hole | 1052437 | 186614 | Inner | No | 2.1 | 3.1 | 13.66 | 0.00 | 0.71 | 4.02 |
| Federated States of Micronesia | Yap | YAP-6 | Gabach Channel | 1049852 | 183837 | Channel | No | 0.9 | 5.2 | 16.44 | 1.33 | 2.45 | 3.57 |
| Federated States of Micronesia | Yap | YAP-7 | Garim | 1045326 | 179338 | Outer | No | 1830.4 | 11.6 | 7.39 | N/A | N/A | N/A |
| Federated States of Micronesia | Yap | YAP-8 | Pelak Channel | 1053411 | 190363 | Channel | No | 1.8 | 10.7 | 13.78 | 1.14 | 1.64 | 5.02 |
| Federated States of Micronesia | Yap | Yap-15 | Rumong outer reef | 1067126 | 186067 | Outer | No | 1064.4 | 16.26 | 11.02 | N/A | N/A | N/A |
| Federated States of Micronesia | Yap | Yap-13 | Af outer reef | 1050819 | 187756 | Outer | Yes | 1647.0 | 7.1 | 10.44 | N/A | N/A | N/A |
| Republic of the Marshall Islands | Namdrik | NAM-1 | Nam-1 | 618775 | 178446 | Outer | No | 253.3 | 1.6 | 15.66 | 0.00 | 0.46 | 1.02 |
| Republic of the Marshall Islands | Namdrik | NAM-2 | Nam-2 | 618469 | 180888 | Outer | No | 785.2 | 3.9 | 11.12 | 0.00 | 0.30 | -0.04 |
| Republic of the Marshall Islands | Namdrik | NAM-3 | Nam-3 | 623866 | 180412 | Outer | No | 2270.6 | 9.1 | 3.17 | 0.00 | 0.74 | -0.66 |
| Republic of the Marshall Islands | Namdrik | NAM-4 | Nam-4 | 623966 | 177201 | Outer | No | 63.6 | 4.6 | 12.39 | N/A | N/A | -0.19 |
| Republic of the Marshall Islands | Namdrik | NAM-5 | Nam-6 | 621759 | 182159 | Outer | No | 2659.0 | 8.3 | 3.03 | 0.00 | 0.24 | 0.32 |
| Republic of the Marshall Islands | Namdrik | NAM-6 | Nam-7 | 618506 | 180399 | Outer | No | 288.9 | 3.5 | 13.14 | 0.00 | 0.25 | 0.73 |

| Island | site | benthic ratio | coral evenness (Shannon H) | coral cover | macroalgae cover |
|---|---|---|---|---|---|
| Aguijan | CNMI-1 | 1.29 | 2.29 | 35.28 | 2.72 |
| Rota | CNMI-10 | 0.50 | 1.74 | 9.04 | 20.34 |
| Rota | CNMI-11 | 0.86 | 1.03 | 9.14 | 20.39 |
| Rota | CNMI-12 | 0.42 | 1.92 | 6.73 | 13.36 |
| Rota | CNMI-13 | 0.46 | 1.84 | 6.32 | 7.17 |
| Rota | CNMI-14 | 0.22 | 1.75 | 5.64 | 6.73 |
| Saipan | CNMI-2 | 1.27 | 2.48 | 36.60 | 6.00 |
| Saipan | CNMI-3 | 0.79 | 2.23 | 30.32 | 1.79 |
| Saipan | CNMI-4 | 0.31 | 2.06 | 5.76 | 6.46 |
| Saipan | CNMI-5 | 1.74 | 2.16 | 24.28 | 3.91 |
| Saipan | CNMI-6 | 1.55 | 2.40 | 24.06 | 6.80 |
| Saipan | CNMI-7 | 1.20 | 2.61 | 38.39 | 0.74 |
| Saipan | CNMI-8 | 0.88 | 2.02 | 21.46 | 6.40 |
| Tinian | CNMI-9 | 1.60 | 2.38 | 35.36 | 2.16 |
| Chuuk | CHK-1 | 1.00 | 0.77 | 31.04 | 24.30 |
| Chuuk | CHK-10 | 1.71 | 1.50 | 38.52 | 17.73 |
| Chuuk | CHK-11 | 0.79 | 1.05 | 19.49 | 31.38 |
| Chuuk | CHK-12 | 2.07 | 1.70 | 35.10 | 15.92 |
| Chuuk | CHK-13 | 1.20 | 1.25 | 27.36 | 9.52 |
| Chuuk | CHK-14 | 2.48 | 0.42 | 32.82 | 5.31 |
| Chuuk | CHK-15 | 0.51 | 0.59 | 4.08 | 39.97 |
| Chuuk | CHK-16 | 1.71 | 1.20 | 30.42 | 6.42 |
| Chuuk | CHK-17 | 1.43 | 1.58 | 32.40 | 18.32 |
| Chuuk | CHK-18 | 1.88 | 1.25 | 24.16 | 4.65 |
| Chuuk | CHK-19 | 2.00 | 1.26 | 28.75 | 19.67 |
| Chuuk | CHK-2 | 1.44 | 0.66 | 26.48 | 17.78 |
| Chuuk | CHK-3 | 2.27 | 0.53 | 27.62 | 12.90 |
| Chuuk | CHK-4 | 1.31 | 0.96 | 30.00 | 9.04 |
| Chuuk | CHK-5 | 3.11 | 1.54 | 38.90 | 2.37 |
| Chuuk | CHK-6 | 0.70 | 0.61 | 21.28 | 30.16 |
| Chuuk | CHK-7 | 1.94 | 1.31 | 38.88 | 16.62 |
| Chuuk | CHK-8 | 1.06 | 1.41 | 17.60 | 21.68 |
| Chuuk | CHK-9 | 1.00 | 1.14 | 28.40 | 32.40 |
| Kosrae | KOS-1 | 3.18 | 1.85 | 66.72 | 7.04 |
| Kosrae | KOS-3 | 1.85 | 1.47 | 44.12 | 4.72 |
| Kosrae | KOS-4 | 1.21 | 1.61 | 41.55 | 9.85 |
| Kosrae | KOS-5 | 0.76 | 2.18 | 16.03 | 6.33 |
| Kosrae | KOS-6 | 2.28 | 1.65 | 51.04 | 3.20 |
| Kosrae | KOS-7 | 0.56 | 2.06 | 20.64 | 24.48 |
| Kosrae | KOS-2 | 2.56 | 1.97 | 62.76 | 9.69 |
| Kosrae | KOS-8 | 2.43 | 1.73 | 48.00 | 2.40 |
| Kosrae | KOS-9 | 1.86 | 2.20 | 46.03 | 5.52 |

| Island | site | benthic ratio | coral evenness (Shannon H) | coral cover | macroalgae cover |
|---|---|---|---|---|---|
| Kosrae | KOS-10 | 1.92 | 1.96 | 50.24 | 3.84 |
| Pohnpei | PNI-1 | 3.33 | 1.42 | 44.24 | 0.00 |
| Pohnpei | PNI-9 | 1.91 | 1.49 | 42.46 | 0.64 |
| Pohnpei | PNI-10 | 2.50 | 1.26 | 15.68 | 15.92 |
| Pohnpei | PNI-11 | 0.50 | 1.44 | 23.50 | 1.92 |
| Pohnpei | PNI-2 | 1.38 | 1.40 | 35.52 | 1.68 |
| Pohnpei | PNI-12 | 1.17 | 1.89 | 25.76 | 1.04 |
| Pohnpei | PNI-13 | 2.88 | 2.42 | 53.99 | 1.92 |
| Pohnpei | PNI-14 | 3.86 | 1.86 | 18.72 | 19.04 |
| Pohnpei | PNI-15 | 3.00 | 1.52 | 8.72 | 19.60 |
| Pohnpei | PNI-16 | 1.76 | 1.32 | 57.68 | 5.92 |
| Pohnpei | PNI-17 | 1.29 | 1.74 | 23.60 | 9.36 |
| Pohnpei | PNI-3 | 3.72 | 1.51 | 39.28 | 0.72 |
| Pohnpei | PNI-4 | 1.30 | 1.65 | 8.53 | 29.44 |
| Pohnpei | PNI-5 | 1.97 | 1.84 | 12.80 | 0.48 |
| Pohnpei | PNI-6 | 1.94 | 1.48 | 21.76 | 6.56 |
| Pohnpei | PNI-7 | 2.85 | 1.60 | 36.72 | 2.88 |
| Pohnpei | PNI-8 | 4.63 | 1.64 | 52.88 | 0.08 |
| Yap | YAP-1 | 3.60 | 1.87 | 49.04 | 0.64 |
| Yap | YAP-9 | 5.17 | 1.73 | 61.76 | 0.00 |
| Yap | YAP-10 | 3.14 | 1.06 | 63.20 | 0.00 |
| Yap | YAP-11 | 3.39 | 1.14 | 54.48 | 0.00 |
| Yap | YAP-12 | 2.58 | 2.25 | 25.04 | 0.00 |
| Yap | YAP-14 | 5.65 | 1.63 | 54.16 | 0.08 |
| Yap | YAP-2 | 5.75 | 1.59 | 65.52 | 1.36 |
| Yap | YAP-3 | 5.51 | 0.82 | 64.16 | 0.00 |
| Yap | YAP-4 | 1.95 | 2.21 | 31.04 | 0.32 |
| Yap | YAP-5 | 4.15 | 0.58 | 43.84 | 0.08 |
| Yap | YAP-6 | 2.60 | 0.99 | 48.48 | 3.44 |
| Yap | YAP-7 | 2.55 | 1.80 | 29.68 | 0.48 |
| Yap | YAP-8 | 3.00 | 1.96 | 49.60 | 0.00 |
| Yap | Yap-15 | 3.59 | 1.62 | 30.53 | 0.17 |
| Yap | Yap-13 | 1.38 | 1.95 | 12.16 | 0.00 |
| Namdrik | NAM-1 | 5.29 | 1.86 | 48.24 | 10.08 |
| Namdrik | NAM-2 | 5.12 | 2.10 | 35.68 | 4.88 |
| Namdrik | NAM-3 | 13.78 | 1.37 | 58.53 | 1.06 |
| Namdrik | NAM-4 | 2.87 | 1.78 | 37.71 | 21.61 |
| Namdrik | NAM-5 | 9.43 | 1.79 | 65.02 | 4.81 |
| Namdrik | NAM-6 | 4.91 | 2.06 | 28.42 | 11.50 |

| Island | Site | fish assem size (cm) | fish ass biomass (kg/SPC) | predator biomass (kg/SPC) | fish heterogeneity (% dissimilarity) | fish evenness (Shannon H) |
|---|---|---|---|---|---|---|
| Aguijan | CNMI-1 | 15.98 | 2.71 | 0.51 | 45.81 | 2.80 |
| Rota | CNMI-10 | 11.98 | 0.95 | 0.07 | 47.56 | 2.21 |
| Rota | CNMI-11 | 14.23 | 7.44 | 0.12 | 54.34 | 0.78 |
| Rota | CNMI-12 | 14.69 | 2.57 | 1.26 | 45.31 | 2.23 |
| Rota | CNMI-13 | 13.44 | 1.78 | 0.03 | 51.49 | 2.71 |
| Rota | CNMI-14 | 11.84 | 1.00 | 0.04 | 41.09 | 1.88 |
| Saipan | CNMI-2 | 14.24 | 2.27 | 0.11 | 43.26 | 2.62 |
| Saipan | CNMI-3 | 10.15 | 0.51 | 0.00 | 41.52 | 1.95 |
| Saipan | CNMI-4 | 10.02 | 0.84 | 0.68 | 38.92 | 1.80 |
| Saipan | CNMI-5 | 11.02 | 2.44 | 0.05 | 41.95 | 2.40 |
| Saipan | CNMI-6 | 8.92 | 0.42 | 0.00 | 32.87 | 1.66 |
| Saipan | CNMI-7 | 14.60 | 0.90 | 0.06 | 30.04 | 0.98 |
| Saipan | CNMI-8 | 10.91 | 2.80 | 0.10 | 38.23 | 2.22 |
| Tinian | CNMI-9 | 13.67 | 0.78 | 0.01 | 38.75 | 1.56 |
| Chuuk | CHK-1 | 12.36 | 1.32 | 2.22 | 47.18 | 2.09 |
| Chuuk | CHK-10 | 13.18 | 2.89 | 5.69 | 51.18 | 1.90 |
| Chuuk | CHK-11 | 21.64 | 10.91 | 4.36 | 49.01 | 2.79 |
| Chuuk | CHK-12 | 22.84 | 16.67 | 82.01 | 36.16 | 2.39 |
| Chuuk | CHK-13 | 18.70 | 5.75 | 3.01 | 52.63 | 2.73 |
| Chuuk | CHK-14 | 16.60 | 4.04 | 0.10 | 48.50 | 2.68 |
| Chuuk | CHK-15 | 14.72 | 4.73 | 2.58 | 50.60 | 2.56 |
| Chuuk | CHK-16 | 14.41 | 2.01 | 0.01 | 57.93 | 2.42 |
| Chuuk | CHK-17 | 18.75 | 9.43 | 68.23 | 34.43 | 1.83 |
| Chuuk | CHK-18 | 14.77 | 4.04 | 3.99 | 49.84 | 2.22 |
| Chuuk | CHK-19 | 19.22 | 8.48 | 29.98 | 51.31 | 2.27 |
| Chuuk | CHK-2 | 9.54 | 0.45 | 0.00 | 36.21 | 1.81 |
| Chuuk | CHK-3 | 14.83 | 3.47 | 0.18 | 53.59 | 2.64 |
| Chuuk | CHK-4 | 12.49 | 1.19 | 0.97 | 46.27 | 1.98 |
| Chuuk | CHK-5 | 18.87 | 6.66 | 45.83 | 17.11 | 2.16 |
| Chuuk | CHK-6 | 9.76 | 0.48 | 0.00 | 34.04 | 1.66 |
| Chuuk | CHK-7 | 13.17 | 3.86 | 9.08 | 56.31 | 2.11 |
| Chuuk | CHK-8 | 14.25 | 2.39 | 1.41 | 59.59 | 2.43 |
| Chuuk | CHK-9 | 17.01 | 5.78 | 19.44 | 47.36 | 2.22 |
| Kosrae | KOS-1 | 16.87 | 3.02 | 0.07 | 47.95 | 2.69 |
| Kosrae | KOS-3 | 15.45 | 4.07 | 0.03 | 34.56 | 2.64 |
| Kosrae | KOS-4 | 16.83 | 4.04 | 0.03 | 40.99 | 2.57 |
| Kosrae | KOS-5 | 15.52 | 3.11 | 0.27 | 47.24 | 3.11 |
| Kosrae | KOS-6 | 15.63 | 4.01 | 0.08 | 39.51 | 2.76 |
| Kosrae | KOS-7 | 14.11 | 2.50 | 0.05 | 39.31 | 2.81 |
| Kosrae | KOS-2 | 16.47 | 3.14 | 0.16 | 46.35 | 3.03 |
| Kosrae | KOS-8 | 17.82 | 6.79 | 0.25 | 46.75 | 2.82 |
| Kosrae | KOS-9 | 16.29 | 4.06 | 0.02 | 42.90 | 2.90 |
| Kosrae | KOS-10 | 15.23 | 1.99 | 0.04 | 39.70 | 2.80 |
| Pohnpei | PNI-1 | 11.97 | 1.54 | 0.01 | 42.67 | 2.54 |
| Pohnpei | PNI-9 | 15.89 | 2.76 | 0.38 | 39.43 | 2.65 |
| Pohnpei | PNI-10 | 17.83 | 6.36 | 0.28 | 43.07 | 2.81 |
| Pohnpei | PNI-11 | 14.31 | 1.72 | 0.17 | 38.85 | 2.57 |
| Pohnpei | PNI-2 | 12.48 | 1.84 | 1.22 | 50.61 | 2.70 |
| Pohnpei | PNI-12 | 17.60 | 3.20 | 0.43 | 50.54 | 3.13 |
| Pohnpei | PNI-13 | 15.00 | 1.64 | 0.00 | 41.72 | 2.46 |
| Pohnpei | PNI-14 | 17.44 | 12.31 | 0.14 | 38.49 | 2.46 |
| Pohnpei | PNI-15 | 21.94 | 34.70 | 1.71 | 42.22 | 2.74 |
| Pohnpei | PNI-16 | 19.85 | 7.92 | 0.40 | 51.18 | 2.62 |
| Pohnpei | PNI-17 | 17.79 | 8.73 | 0.51 | 40.46 | 2.71 |
| Pohnpei | PNI-3 | 13.08 | 1.45 | 0.09 | 48.47 | 2.77 |
| Pohnpei | PNI-4 | 11.28 | 2.11 | 0.02 | 45.30 | 2.45 |
| Pohnpei | PNI-5 | 12.16 | 2.77 | 0.04 | 33.09 | 2.46 |
| Pohnpei | PNI-6 | 12.72 | 5.21 | 0.18 | 44.96 | 2.97 |
| Pohnpei | PNI-7 | 13.50 | 2.68 | 0.32 | 49.33 | 2.05 |
| Pohnpei | PNI-8 | 12.11 | 1.32 | 0.04 | 46.68 | 2.76 |
| Yap | YAP-1 | 11.36 | 2.14 | 0.14 | 43.65 | 2.08 |
| Yap | YAP-9 | 14.00 | 1.80 | 0.22 | 55.21 | 2.22 |
| Yap | YAP-10 | 10.33 | 0.65 | 0.00 | 37.85 | 1.75 |
| Yap | YAP-11 | 7.86 | 0.37 | 0.13 | 55.85 | 1.73 |
| Yap | YAP-12 | 11.22 | 1.75 | 0.39 | 54.83 | 2.35 |
| Yap | YAP-14 | 14.29 | 2.02 | 0.93 | 50.00 | 2.13 |
| Yap | YAP-2 | 13.15 | 1.46 | 0.06 | 40.56 | 2.33 |
| Yap | YAP-3 | 9.85 | 0.34 | 0.02 | 53.40 | 2.05 |

| Island | Site | fish assem size (cm) | fish ass biomass (kg/SPC) | predator biomass (kg/SPC) | fish heterogeneity (% dissimilarity) | fish evenness (Shannon H) |
|---|---|---|---|---|---|---|
| Yap | YAP-4 | 13.10 | 2.15 | 0.80 | 47.19 | 1.96 |
| Yap | YAP-5 | 9.71 | 0.51 | 0.07 | 36.02 | 1.73 |
| Yap | YAP-6 | 12.87 | 3.66 | 0.40 | 52.43 | 2.58 |
| Yap | YAP-7 | 13.11 | 4.79 | 1.23 | 48.72 | 2.66 |
| Yap | YAP-8 | 10.87 | 1.09 | 0.40 | 34.45 | 1.90 |
| Yap | Yap-15 | 14.98 | 2.52 | 0.36 | 57.59 | 2.93 |
| Yap | Yap-13 | 13.28 | 4.81 | 0.48 | 46.86 | 2.93 |
| Namdrik | NAM-1 | 19.24 | 4.23 | 1.82 | 53.13 | 2.46 |
| Namdrik | NAM-2 | 19.48 | 6.27 | 2.78 | 56.19 | 2.89 |
| Namdrik | NAM-3 | 19.04 | 5.77 | 7.52 | 48.34 | 2.74 |
| Namdrik | NAM-4 | 22.41 | 8.58 | 4.75 | 52.60 | 2.61 |
| Namdrik | NAM-5 | 17.24 | 3.75 | 1.56 | 49.72 | 2.91 |
| Namdrik | NAM-6 | 16.46 | 2.95 | 5.29 | 44.79 | 1.99 |

| Island | Site | coral skewness | coral heterogeneity (% dissimilarity) | species richness | coral evenness (Shannon H) |
|---|---|---|---|---|---|
| Aguijan | CNMI-1 | 2.53 | 65.91 | 6.50 | 3.30 |
| Rota | CNMI-10 | 4.37 | 54.56 | 6.94 | 2.53 |
| Rota | CNMI-11 | 2.72 | 50.84 | 5.27 | 1.22 |
| Rota | CNMI-12 | 1.73 | 59.93 | 5.88 | 2.63 |
| Rota | CNMI-13 | 2.43 | 62.13 | 4.38 | 2.41 |
| Rota | CNMI-14 | 1.68 | 61.10 | 5.88 | 2.82 |
| Saipan | CNMI-2 | 4.14 | 56.79 | 10.00 | 3.14 |
| Saipan | CNMI-3 | 2.34 | 56.78 | 8.75 | 3.04 |
| Saipan | CNMI-4 | 2.74 | 58.27 | 7.19 | 2.91 |
| Saipan | CNMI-5 | 2.99 | 60.25 | 8.56 | 2.96 |
| Saipan | CNMI-6 | 3.91 | 64.19 | 5.81 | 3.10 |
| Saipan | CNMI-7 | 3.74 | 61.02 | 6.56 | 2.88 |
| Saipan | CNMI-8 | 2.60 | 59.50 | 7.44 | 3.00 |
| Tinian | CNMI-9 | 2.68 | 61.90 | 8.13 | 3.30 |
| Chuuk | CHK-1 | 3.86 | 54.80 | 4.20 | 1.70 |
| Chuuk | CHK-10 | 3.17 | 45.94 | 7.90 | 2.00 |
| Chuuk | CHK-11 | NA | NA | NA | NA |
| Chuuk | CHK-12 | NA | NA | NA | NA |
| Chuuk | CHK-13 | NA | NA | NA | NA |
| Chuuk | CHK-14 | NA | NA | NA | NA |
| Chuuk | CHK-15 | NA | NA | NA | NA |
| Chuuk | CHK-16 | NA | NA | NA | NA |
| Chuuk | CHK-17 | NA | NA | NA | NA |
| Chuuk | CHK-18 | NA | NA | NA | NA |
| Chuuk | CHK-19 | NA | NA | NA | NA |
| Chuuk | CHK-2 | 2.30 | 55.06 | 2.40 | 1.22 |
| Chuuk | CHK-3 | 3.60 | 50.99 | 6.80 | 1.45 |
| Chuuk | CHK-4 | 6.60 | 60.99 | 7.20 | 1.99 |
| Chuuk | CHK-5 | 4.12 | 51.77 | 9.20 | 2.31 |
| Chuuk | CHK-6 | 4.20 | 50.33 | 2.50 | 1.15 |
| Chuuk | CHK-7 | 2.54 | 54.25 | 6.00 | 2.09 |
| Chuuk | CHK-8 | 3.30 | 61.20 | 4.40 | 1.81 |
| Chuuk | CHK-9 | 3.00 | 53.39 | 2.60 | 1.36 |
| Kosrae | KOS-1 | 3.82 | 57.66 | 5.40 | 2.57 |
| Kosrae | KOS-3 | 2.89 | 61.20 | 3.50 | 1.89 |
| Kosrae | KOS-4 | 5.29 | 48.75 | 8.80 | 2.28 |
| Kosrae | KOS-5 | 4.37 | 53.98 | 9.10 | 2.48 |
| Kosrae | KOS-6 | 4.10 | 58.74 | 4.90 | 2.34 |
| Kosrae | KOS-7 | 3.93 | 61.17 | 5.60 | 2.33 |
| Kosrae | KOS-2 | 3.61 | 58.06 | 7.40 | 2.65 |
| Kosrae | KOS-8 | 1.99 | 50.21 | 11.30 | 2.83 |
| Kosrae | KOS-9 | 3.66 | 62.09 | 5.10 | 2.74 |
| Kosrae | KOS-10 | 3.51 | 52.82 | 6.60 | 2.33 |
| Pohnpei | PNI-1 | 3.32 | 51.72 | 5.70 | 1.66 |
| Pohnpei | PNI-9 | 4.40 | 29.60 | 6.20 | 1.84 |
| Pohnpei | PNI-10 | 4.65 | 48.99 | 7.90 | 1.73 |
| Pohnpei | PNI-11 | 2.58 | 48.19 | 4.70 | 1.75 |
| Pohnpei | PNI-2 | 4.50 | 52.13 | 5.30 | 1.49 |
| Pohnpei | PNI-12 | 4.84 | 53.27 | 5.40 | 1.61 |
| Pohnpei | PNI-13 | 2.55 | 58.86 | 9.90 | 3.01 |
| Pohnpei | PNI-14 | 2.58 | 60.11 | 5.50 | 2.26 |

| Island | Site | coral skewness | coral heterogeneity (% dissimilarity) | species richness | coral evenness (Shannon H) |
|---|---|---|---|---|---|
| Pohnpei | PNI-15 | 1.37 | 61.38 | 6.60 | 2.61 |
| Pohnpei | PNI-16 | 2.39 | 29.16 | 3.90 | 1.34 |
| Pohnpei | PNI-17 | 2.50 | 56.73 | 7.40 | 2.08 |
| Pohnpei | PNI-3 | 4.73 | 40.04 | 3.60 | 1.20 |
| Pohnpei | PNI-4 | 3.20 | 50.00 | 5.60 | 1.63 |
| Pohnpei | PNI-5 | 2.79 | 46.61 | 9.70 | 2.15 |
| Pohnpei | PNI-6 | 2.70 | 42.39 | 8.80 | 1.48 |
| Pohnpei | PNI-7 | 5.16 | 44.84 | 7.20 | 1.60 |
| Pohnpei | PNI-8 | 4.37 | 40.34 | 6.70 | 1.68 |
| Yap | YAP-1 | 1.45 | 51.71 | 8.20 | 2.60 |
| Yap | YAP-9 | 4.45 | 56.56 | 7.25 | 2.48 |
| Yap | YAP-10 | 4.92 | 45.70 | 6.50 | 1.68 |
| Yap | YAP-11 | 3.80 | 48.91 | 4.13 | 1.67 |
| Yap | YAP-12 | 4.50 | 55.02 | 9.50 | 2.20 |
| Yap | YAP-14 | 6.54 | 51.73 | 9.40 | 1.95 |
| Yap | YAP-2 | 3.60 | 54.22 | 5.75 | 2.36 |
| Yap | YAP-3 | 4.33 | 39.53 | 3.88 | 1.16 |
| Yap | YAP-4 | 3.69 | 55.59 | 11.00 | 2.63 |
| Yap | YAP-5 | 2.44 | 34.22 | 2.13 | 1.04 |
| Yap | YAP-6 | 2.78 | 24.91 | 4.50 | 1.04 |
| Yap | YAP-7 | 3.56 | 53.34 | 9.63 | 2.16 |
| Yap | YAP-8 | 3.47 | 49.78 | 5.25 | 2.11 |
| Yap | Yap-15 | 4.46 | 35.13 | 12.20 | 1.80 |
| Yap | Yap-13 | 2.29 | 58.15 | 8.00 | 2.56 |
| Namdrik | NAM-1 | 2.99 | 50.72 | 5.80 | 1.87 |
| Namdrik | NAM-2 | 3.19 | 56.51 | 4.20 | 2.29 |
| Namdrik | NAM-3 | 2.03 | 42.39 | 7.60 | 2.39 |
| Namdrik | NAM-4 | 4.33 | 52.56 | 5.60 | 1.75 |
| Namdrik | NAM-5 | 2.44 | 53.47 | 8.60 | 2.20 |
| Namdrik | NAM-6 | 1.89 | 49.62 | 4.80 | 2.61 |
