# Supplementary material for: The Micronesia Challenge: Assessing the Relative Contribution of Stressors on Coral Reefs to Facilitate Science-to-Management Feedback
Source: PLoS One. 2015 Jun 18;10(6):e0130823. doi: 10.1371/journal.pone.0130823 (PMC4473011; doi:10.1371/journal.pone.0130823)
Supplement: S3 Table — (PDF) [file pone.0130823.s006.pdf]

JURISDICTION	REEF-FISHERY REGULATIONS	LAND-BASED DISCHARGE REGULATIONS	GAPS
**YAP, FSM**	No reef-fish export can exceed 50 lbs per person/event; Fishing with chemicals and explosives prohibited; 3-inch minimum gillnet size; no-take MPA; All laws subject to exclusion for practices in accordance with tradition and custom	No dumping of trash or any pollutant in unrestricted areas without EPA approval; Earthmoving permit required for development; Environmental impact assessments required for major development pending EPA decision; All laws subject to exclusion for practices in accordance with tradition and custom	Export policies; Controls on night free-dive fishing with flashlights; Protection during spawning periods, size-limits; subsistence catch quota's; SCUBA fishing policies; pollution control for development prior to permitting system; master plan for non-point source watershed discharge; sewage and wastewater regulations; Overlap between roles of regulatory agencies and traditional cultural practices
**CHUUK, FSM**	Ban on *Bolbometopon muricatum* and *Cheilinus undulatus* export; seasonal ban on all grouper sales from January to March; no-take MPA	Earthmoving and septic/sewer permit required for development; no septic or piggeries 50 ft. from wells, rivers, or coastlines	Reef fisheries export policies; controls on night free-dive fishing with flashlights; protection during spawning periods besides groupers, size-limits; subsistence catch quota's; controls on live reef fish trade; SCUBA fishing policies; enforcement; pollution control for development prior to permitting system; master plan for non-point source watershed discharge
**POHNPEI, FSM**	**New regulations 2014**. Complete ban on *Bolbometopon muricatum* and *Cheilinus undulatus* fishing or sale; seasonal ban on *Plectropomus areolatus* January to May, all other grouper from February to April; SCUBA fishing ban; no-take MPA; 4-inch minimum gillnet size; size-class regulations for 9 food fish*[1]	Permits required for all new residential and commercial development; household septic/sewer system requirements; no dumping of trash or any pollutant in unrestricted areas; Piggeries set back 50 ft. from houses and waterbodies, and must have septic systems	Reef fisheries export policies; controls on night free-dive fishing with flashlights; catch quota's; pollution control for development prior to permitting system; master plan for non-point source watershed discharge
**KOSRAE, FSM**	SCUBA fishing ban; no-take MPA; *Bolbometopon muricatum* harvesting ban; prohibition of any destructive fishing methods;	Permits required for all new residential and commercial development since 1992; household septic/sewer system requirements; no dumping of trash or any pollutant in unrestricted	Controls on night free-dive fishing with flashlights; Protection during spawning periods, size-limits; subsistence catch quota's; pollution control for development prior to

		areas; Piggeries set back 50 ft. from houses and waterbodies, and must have septic systems	permitting system; master plan for non-point source watershed discharge
**NAMDRIK, RMI**	License requirements for commercial fishing or regulated species; no-take MPA's; prohibition of any destructive fishing methods; size and seasonal restrictions for key species, minimum net mesh sizes	No dumping of trash or any pollutant in unrestricted areas; piggery location and waste ordinances; household septic tank requirements	Enforcement; community education; capacity; funding; plan for non-point source watershed discharge
**CNMI**	Gill-net ban for drag nets, surround nets, seines and trap nets, with limited cultural exceptions; SCUBA fishing ban; no-take MPA's; no bleach, cyanide, or '*derris*'[*2]; no explosives; no traps; annual commercial catch limits for major reef fish species and families[*3]	Permits required for all new residential and commercial development, including sewer/septic since 1983; Permits required for all point-source discharges since 1986; Permitting revision in 1993 include controlling non-point source pollution for new construction	Controls on night free-dive fishing with flashlights; Protection during spawning periods, size-limits; subsistence catch quota's; enforcement; pollution control for development prior to permitting system; updated master plan for non-point source watershed discharge

[*1]14-inches for *Naso unicornis*, all groupers, all trevally and jacks, all sweetlips, *Chlorurus microrhinos*; 10-inches for *Lutjanus gibbus*, all rudderfish, *Hipposcarus longiceps*; 24-inches for *Epinephelus fuscoguttatus*

[*2]Local term for plant root with rotenone fish poison

[*3]Annual catch limits that vary based upon US National Marine Fisheries Service analyses, adopted by the Western Pacific Fisheries Management Council for Acanthuridae, Lutjanidae, *Selar crumenophthalmus*, Carangidae, Lethrinidae, Scaridae, Serranidae, Holocentridae, Mugilidae, *Bolbometopon muricatum*, *Cheilinus undulatus*, Carcharhinidae, and all others combined
